# Inducing Receptor Degradation as a Novel Approach to Target CC Chemokine Receptor 2 (CCR2)

**DOI:** 10.3390/ijms25168984

**Published:** 2024-08-18

**Authors:** Natalia V. Ortiz Zacarías, Sascha Röth, Jeremy D. Broekhuis, Daan van der Es, Kevin Moreau, Laura H. Heitman

**Affiliations:** 1 Division of Medicinal Chemistry, Leiden Academic Centre for Drug Research (LACDR), Leiden University, 2333 CC Leiden, The Netherlands; jeremybroekhuis@outlook.com (J.D.B.); d.van.der.es@lacdr.leidenuniv.nl (D.v.d.E.); l.h.heitman@lacdr.leidenuniv.nl (L.H.H.); 2 Safety Innovation and PROTAC Safety, Clinical Pharmacology & Safety Sciences, R&D, AstraZeneca, Cambridge CB2 0AA, UK; sascha.roth@astrazeneca.com (S.R.); kevin.moreau@astrazeneca.com (K.M.); 3 Oncode Institute, 2333 CC Leiden, The Netherlands

**Keywords:** targeted degradation, GPCRs, CCR2, HaloTag

## Abstract

CC chemokine receptor 2 (CCR2) has been linked to many inflammatory and immune diseases, making it a relevant drug target. Yet, all CCR2 antagonists developed so far have failed in clinical trials; thus, novel strategies are needed to target this receptor. Targeted protein degradation represents a novel approach to inhibit protein function by hijacking the cellular degradation machinery, such as the proteasome, to degrade the protein of interest. Here, we aimed to determine the amenability of CCR2 to chemically induced degradation by using a CCR2 fusion protein containing a HaloTag7 and HiBiT tag (CCR2-HaloTag-HiBiT). After characterization of the CCR2 construct, we used luminescence-based assays and immunofluorescence to quantify CCR2 levels, as well as a label-free, phenotypic assay to investigate the functional effect of CCR2 degradation. Treatment with HaloPROTAC3, which selectively degrades HaloTag fusion proteins, led to concentration- and time-dependent degradation of CCR2-HaloTag-HiBiT. HaloPROTAC3 induced degradation via the proteasome, as degradation was fully blocked with proteasomal inhibitors. Finally, functional assays showed that degradation of CCR2-HaloTag-HiBiT leads to a reduced functional response after agonist stimulation. Overall, our results indicate that CCR2 is amenable to targeted degradation, paving the way for the future development of CCR2 chemical degraders.

## 1. Introduction

GPCRs represent one of the most targeted protein classes to date, accounting for more than 30% of approved drugs on the market. Yet, these drugs only target a minority of GPCRs, while many remain ‘undruggable’ [1,2]. One such GPCR is the CC Chemokine receptor 2 (CCR2), which is highly expressed in immune cells, such as monocytes, macrophages and natural killer cells [3]. Activation of CCR2 by its endogenous ligands, mainly the chemokine CCL2, results in a variety of signaling pathways that control the migration of immune cells into sites of inflammation, as well as their differentiation and survival [4]. Dysregulation of the CCL2-CCR2 signaling axis has been linked to many inflammatory and immune diseases, including atherosclerosis [5,6] and cancer [7]. Over the years, many CCR2 antagonists have been pursued as clinical candidates; however, none of them have made it to the clinic yet [8]. Thus, novel strategies are needed to successfully target this receptor.

Over the last few years, targeted protein degradation (TPD) has arisen as a novel therapeutic strategy to inhibit protein function by harnessing different native cellular degradation systems [9]. PROteolysis-TArgeting Chimeras (PROTACs) represent one of the pioneering TPD technologies that make use of the ubiquitin-proteasome system (UPS) to induce degradation of the target protein. PROTACs are heterobifunctional molecules composed of two linked ligands: one that binds to the protein of interest and another one that binds to a ubiquitin E3 ligase [10]. As part of the UPS system, E3 ligases catalyze the attachment of ubiquitin molecules to lysine residues of a target protein, which results in a variety of cellular fates, including proteasomal degradation of the target protein. While the pool of over 600 E3 ligases provides highly specific E3–substrate pairings, PROTACs can induce non-natural ternary complexes with both the E3 ligase and the target, resulting in target polyubiquitination and degradation [10]. Although PROTAC development has been focused on cytosolic and nuclear target proteins, there are now some examples that suggest its feasibility in other types of proteins, including integral membrane proteins such as receptor tyrosine kinases [11], solute carrier transporters [12], and G protein-coupled receptors (GPCRs) [13,14,15] (also extensively reviewed in [16]). Compared to traditional inhibitors, PROTACs may provide several clinical benefits: a longer therapeutic effect due to complete removal of the target protein; lower doses needed due to their catalytic mechanism of action; and potential for tissue selectivity by engaging differentially expressed E3 ligases [17,18].

GPCRs are usually targeted with agonists or antagonists that bind to a canonical binding site, the so-called ‘orthosteric’ binding site, located at the extracellular side of the receptor. However, a variety of distinct binding sites have been reported in literature, including an allosteric binding site located at the intracellular interface of many GPCRs [19,20]. Such an intracellular binding site has also been identified in CCR2, and has been confirmed by X-ray crystallography [21]. As PROTACs need to recruit cytosolic E3 ligases, these intracellular ligands may serve as starting points for the development of GPCR-targeting PROTACs. In this regard, Huber et al. made use of CCR9 intracellular ligands and functionalized them as PROTACs by linking them to a von Hippel-Lindau (VHL) E3 ligase ligand. This resulted in one PROTAC that was able to induce ~30% degradation of CCR9 as quantified in an enzyme-linked immunosorbent assay (ELISA) [15], supporting the validity of this approach for CCR2 and GPCRs in general.

The design and development of PROTACs remains a largely empirical field, with no clear principles to predict degradation efficacy, making it a slow and resource-intensive process [22,23]. In order to circumvent the need to immediately develop PROTACs, as well as to gain information on the target’s degradation amenability, several tag-targeted degrader technologies have been developed. These tag-targeted degraders selectively bind to a specific tag and degrade tag-fusion proteins [24,25]. Different protein tags have been used for these technologies, including HaloTag7 [26], dTAG [27], BromoTag [28], or Nanoluciferase [25]. For example, HaloPROTAC3 contains a chloroalkane moiety—which covalently binds to HaloTag7—linked to the VHL ligand VL285 [26,29]. By recruiting VHL to the HaloTag7-fusion protein, HaloPROTAC3 is able to induce ubiquitination and proteasomal degradation of the tagged-protein.

In this study, we made use of a tag-targeted system to explore the amenability of CCR2 to targeted degradation. To this end, we designed and characterized a CCR2-fusion protein—CCR2-HaloTag-HiBiT—containing HaloTag7 to induce degradation by HaloPROTAC3, and a HiBiT tag [30] to measure protein levels using a previously described luminescence-based assay [31,32,33]. Using a variety of endpoint and real-time assays, we show that HaloPROTAC3 is able to induce degradation of our CCR2-HaloTag-HiBiT fusion protein in a concentration- and time-dependent manner, while this is not observed with a negative control or with traditional CCR2 inhibitors. Furthermore, these assays allowed us to shed light on the degradation pathways involved in HaloPROTAC3-induced degradation of CCR2, by confirming a VHL- and proteasome-dependent mechanism. Finally, we investigated the functional consequence of CCR2 degradation using a label-free, phenotypic whole-cell assay. Overall, our results indicate that CCR2 is amenable to targeted degradation; therefore, the development of CCR2-PROTACs is warranted as a novel strategy to target this receptor, and other GPCRs containing an intracellular binding site.

## 2. Results

### 2.1. Insertion of HaloTag7 to CCR2 Alters Receptor Function but Not Ligand Binding

**Figure 1:**
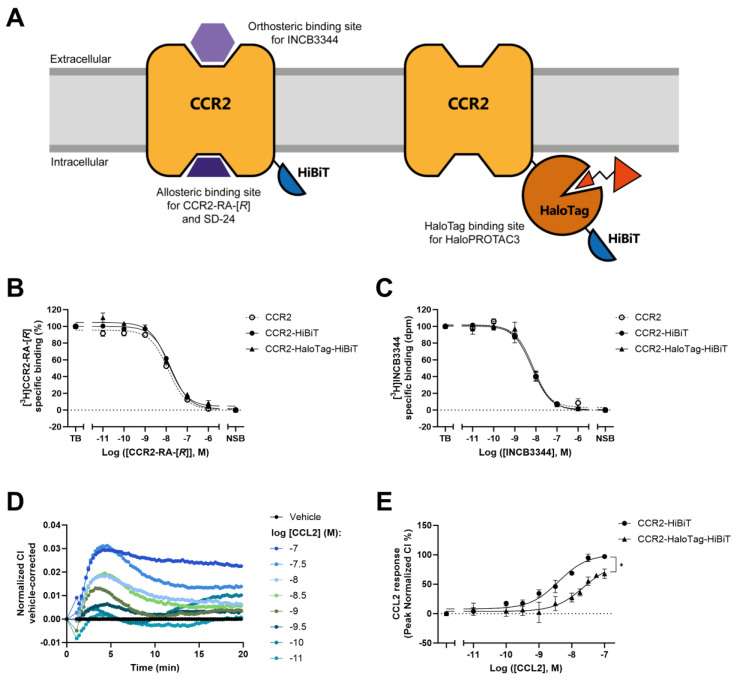
Pharmacological characterization of CCR2 constructs using radioligand binding and functional assays. (**A**) Schematic representation of the two CCR2 constructs designed for this study, CCR2-HiBiT, and CCR2-HaloTag-HiBiT, containing the two tags in the intracellular interface of the receptor. The representation also depicts the binding sites where the different compounds bind. (**B**,**C**) Homologous displacement curves of [^3^H]CCR2-RA-[*R*] (**B**) or [^3^H]INCB3344 (**C**) specific binding by increasing concentrations of non-labeled CCR2-RA-[*R*] or INCB3344, respectively, in membranes from U2OS cells stably expressing CCR2 (referred as CCR2 in legend), or from HEK293T cells transiently transfected with CCR2-HiBiT or CCR2-HaloTag-HiBiT (referred as CCR2-HiBiT and CCR2-HaloTag-HiBiT in legend). Radioligand binding data are shown as mean ± SEM of three independent experiments performed in duplicates. (**D**) Representative vehicle-corrected, normalized Cell Index (CI) traces measured in xCELLigence after stimulation of HEK293T cells transfected with CCR2-HiBiT with vehicle (PBS) or increasing concentrations of CCL2. Data are shown as representative mean CI values of a single experiment performed in duplicate. (**E**) Combined concentration-response curves of CCL2 on HEK293T cells transfected with CCR2-HiBiT or CCR2-HaloTag-HiBiT. CCL2 cellular response was derived from the vehicle-corrected, normalized CI traces and is expressed as the maximum peak response within the first 15–20 min after stimulation. Data are shown as mean ± SEM of at least three independent experiments performed in triplicates. Statistical differences between normalized responses at the highest CCL2 concentrations were analyzed using an unpaired *t*-test analysis: * *p* < 0.05.

To explore tag-targeted degradation of CCR2, we designed two CCR2 fusion proteins containing a C-terminal HaloTag7-HiBiT (CCR2-HaloTag-HiBiT) or only a HiBiT (CCR2-HiBiT) in the intracellular region (Figure 1A). Of note, we used the human CCR2 isoform B (hCCR2B) for the design and expression of the fusion proteins in HEK293T cells. As the addition of the two tags, particularly the 34 kDa HaloTag7, may affect receptor conformation and/or receptor function, we first characterized ligand binding using radioligand binding assays. We performed homologous radioligand binding assays with two previously characterized radioligands: the orthosteric [^3^H]INCB3344, which binds at an extracellular binding site, and the allosteric [^3^H]CCR2-RA-[*R*], which binds at the intracellular interface of CCR2, closer to the location of the two tags (Figure 1A and Figure S1). These assays were performed in membrane preparations from HEK293T cells transiently expressing CCR2-HiBiT or CCR2-HaloTag-HiBiT, as well as in membranes from previously established U2OS cells modified to stably express hCCR2B as control. In order to prevent large differences in expression levels, we chose membrane concentrations (10–20 µg) that resulted in similar radioligand binding for all samples (Figure S2A). Titration of the respective unlabeled compounds leads to a concentration-dependent reduction in radioligand binding. In U2OS membranes containing CCR2, both antagonists displaced their respective radioligands with pIC_50_ values in the range of 7.9 to 8.2 (Figure 1B,C and Table 1), which is in line with previous studies [34,35]. The addition of the HiBiT tag or the much bigger HaloTag7 to the C-terminus of CCR2 did not affect the binding of the two antagonists, as their affinities to these constructs were similar to those measured on wild-type CCR2 (Figure 1B,C and Table 1). The C-terminal location of the tags meant that we also included a second intracellular antagonist, SD-24, belonging to a different chemical scaffold than CCR2-RA-[*R*] (Figure S1). In accordance with the other antagonists, the affinity of SD-24 was comparable among the constructs and wild-type CCR2 (Figure S2B and Table 1).

While ligand binding was not affected by the C-terminal tags, receptor function could still be affected, as signaling effectors such as G proteins and β-arrestin interact with GPCRs via the intracellular region. Thus, we used a label-free, impedance-based cellular assay (xCELLigence) to investigate CCR2 function after stimulation with the endogenous ligand CCL2 [34,36]. This method relies on the real-time measurement of electrical impedance, which is generated by changes in proliferation, morphology, or viability of cells attached to golden electrodes on the bottom of the wells. GPCR-mediated signaling can result in morphological changes, which correspond to changes in electrical impedance, reported as the unitless parameter Cell index (CI) [36,37]. HEK293T cells transiently transfected with CCR2-HiBiT or CCR2-HaloTag-HiBiT were seeded in gold-coated 96-well E-plates and left to proliferate for ~20 h before stimulating with increasing concentrations of CCL2. In addition, control experiments were performed using U2OS cells stably expressing CCR2. In agreement with prior literature using U2OS cells with stable CCR2 expression [34], CCL2 induced a fast concentration-dependent increase in impedance, as shown by the increase in normalized CI values, with a peak response within the first 6 min (Figure S2C) and a potency (EC_50_) of 3 nM (pEC_50_ = 8.6 ± 0.2, Figure S2D). Stimulation of CCR2-HiBiT or CCR2-HaloTag-HiBiT with CCL2 also resulted in a concentration-dependent increase in impedance, with peak normalized CI values within the first 3 to 12 min (Figure 1D). The potency of CCL2 in HEK293T cells expressing CCR2-HiBiT (pEC_50_ = 8.4 ± 0.1; EC_50_ = 4 nM), was very similar to its potency in U2OS cells expressing CCR2. However, CCL2 displayed a lower potency (pEC_50_ = 7.6 ± 0.2; EC_50_ = 29 nM) and lower efficacy (E_max_ = 68 ± 8% versus 97 ± 4%) in CCR2-HaloTag-HiBiT compared to CCR2-HiBiT (Figure 1E). In short, the addition of the small HiBiT tag did not affect CCR2 function, while the bigger HaloTag caused a decrease in potency and efficacy of CCL2-induced CCR2 signaling.

### 2.2. HaloPROTAC3 Induces Degradation of CCR2-HaloTag-HiBiT

**Figure 2:**
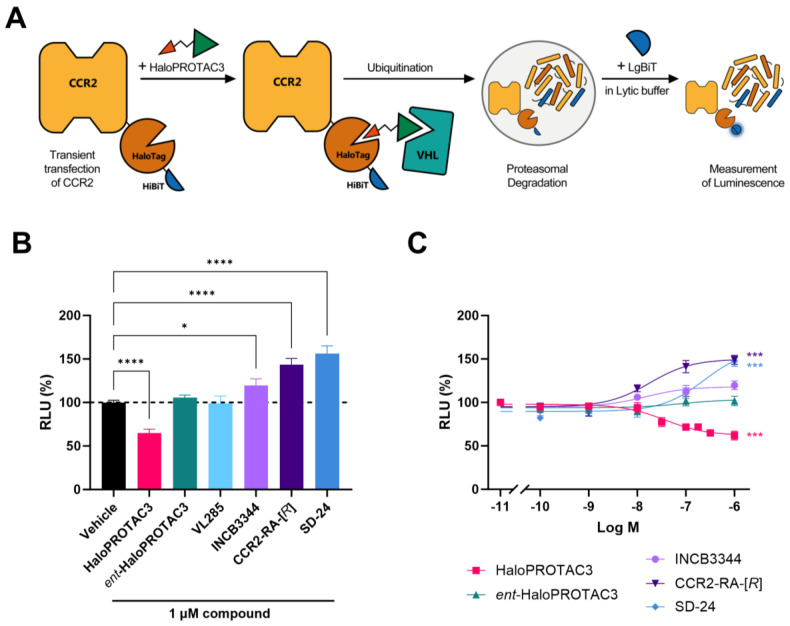
Lytic HiBiT detection assays to measure CCR2 levels after treatment with indicated compounds for 24 h. (**A**) Schematic drawing of the HiBiT lytic assay used to assess CCR2-HaloTag-HiBiT degradation. Assays were performed with HEK293T cells transfected with 5 µg of CCR2-HaloTag HiBiT. Transfected cells were then treated with selected compounds for 24 h before the addition of LgBiT protein in lytic buffer and measurement of luminescence. (**B**,**C**) Relative light units (RLUs) measured after treatment of cells with a single concentration of 1 µM (**B**) or with multiple concentrations (**C**) of the indicated compounds for 24 h. In all cases, RLU values from mock-transfected HEK293T cells were used for baseline-correction, and data were normalized to vehicle control (100%). Data are shown as mean ± SEM of at least three independent experiments performed in triplicates. Statistical differences between normalized RLU values were analyzed using one-way ANOVA with Dunnett’s post-hoc test: * *p* < 0.05, *** *p* < 0.001, **** *p* < 0.0001.

After validating that ligand binding was not affected on the CCR2-HaloTag-HiBiT construct, we aimed to investigate if it was possible to induce its degradation with the commercially available HaloPROTAC3. To determine the expression levels of CCR2 and establish a high-throughput screening method, we made use of the HiBiT tag in the construct, which allowed us to measure protein levels by means of luminescence. To do so, we set up a lytic HiBiT assay (Figure 2A) that relies on the complementation of the HiBiT tag with the membrane-impermeable LgBiT protein. As the tag is located in the C-terminal side of CCR2, a lytic buffer was used to ensure that the LgBiT reaches the HiBiT tag. Transfection of HEK293T with CCR2-HaloTag-HiBiT resulted in a luminescent signal, even at the lowest tested DNA plasmid concentration of 1 µg per 10 cm ø plate. Incrementing the plasmid concentration to 3 µg, 5 µg, or 10 µg per plate led to ~14-, 50-, and 400-fold increase in luminescence, respectively, from ~150 relative light units (RLUs) at the 1 µg of DNA plasmid to ~60,000 RLU at the highest plasmid concentration (Figure S3). Of note, mock-transfected HEK293T cells had only a marginal level of background luminescence.

The tool compound HaloPROTAC3 was designed to induce the degradation of HaloTag fusion proteins by binding both HaloTag7 and VHL [26] (Figure 2A and Figure S1); thus, we aimed to investigate whether this compound was also able to induce degradation of our CCR2-HaloTag-HiBiT construct. As a negative control, we used the enantiomer of HaloPROTAC3, *ent*-HaloPROTAC3, which still binds covalently to the HaloTag but lacks binding to the VHL protein (Figure S1). As an additional control, we used the VHL inhibitor VL285, which corresponds to the VHL-binding ligand from which HaloPROTAC3 originated [26]. 24 h after transfecting HEK293T cells with 5 µg of DNA encoding CCR2-HaloTag-HiBiT, cells were seeded in 96-well plates and treated with 1 µM of HaloPROTAC3, *ent*-HaloPROTAC3, VL285 or vehicle control for another 24 h before measuring luminescence. Treatment with 1 µM of HaloPROTAC3 led to a significant reduction in luminescent signal compared to vehicle control, corresponding to a ~35% decrease in CCR2 levels (Figure 2B), while signals were comparable to vehicle control when cells were treated with *ent*-HaloPROTAC3 or VL285. Of note, the observed decrease in luminescence with HaloPROTAC3 was detected among different plasmid concentrations, ranging from ~30 to 60% degradation of CCR2 (Figure S3). Due to the high luminescent signal and sufficient CCR2 degradation, we continued with 5 µg of plasmid per plate for further assays.

We also tested the orthosteric and allosteric CCR2 antagonists INCB3344, CCR2-RA-[*R*], and SD-24 in this lytic HiBiT assay to investigate whether traditional CCR2 inhibition results in protein degradation. Treatment with 1 µM of these antagonists did not reduce CCR2 levels; instead, all antagonists increased the levels of CCR2 compared to vehicle control. This increase was more pronounced with the intracellular antagonists CCR2-RA-[*R*] and SD-24, which induced a 43% and 56% increase, respectively, in CCR2 levels compared to a 20% increase by INCB3344 (Figure 2B). Next, we determined if the decrease or increase in receptor levels was concentration-dependent or just an artifact of using a high ligand concentration. Increasing concentrations of HaloPROTAC3 led to a concentration-dependent reduction in CCR2 levels, with an apparent degradation potency (DC_50_) of 42 nM (pDC_50_ of 7.4 ± 0.01) and an apparent maximal degradation (D_max_) of 38 ± 5% at 24 h (Figure 2C and Table 2), consistent with our findings in the single-concentration experiment. In line with our previous assays, receptor levels remained unchanged upon titration of *ent*-HaloPROTAC3, while INCB3344, CCR2-RA-[*R*], and SD-24 led to a concentration-dependent increase of CCR2 levels up to 120%, 149%, and 148%, respectively (Figure 2C and Table 2). INCB3344 and CCR2-RA-[*R*] induced CCR2 accumulation with similar apparent potencies (EC_50_) of 6 nM and 15 nM (pEC_50_ of 8.4 ± 0.3 for INCB3344 and 7.8 ± 0.1 for CCR2-RA-[*R*]). In contrast, the potency of SD-24 (pEC_50_ of 6.8 ± 0.1) was >10-fold lower than the potency of the other antagonists (Figure 2C and Table 2).

### 2.3. Real-Time HaloPROTAC3-Induced Degradation of CCR2

Next, we investigated HaloPROTAC3-induced CCR2 degradation over time. As the lytic HiBiT assay allows us to measure luminescence only at a single timepoint, we used HaloPROTAC3 at a concentration that yielded maximal degradation, i.e., 1 µM, and we added it to the cells at different time points ranging from 24 h to 30 min before measuring luminescence. Approximately 20% of CCR2 was degraded after 4 h, while maximum degradation at 24% (similar to 26% after 24 h) was achieved by 6 h (Figure S4). However, the limited amount of timepoints and the small degradation window prevented us from fully characterizing the kinetic profile of HaloPROTAC3-induced degradation.

**Figure 3:**
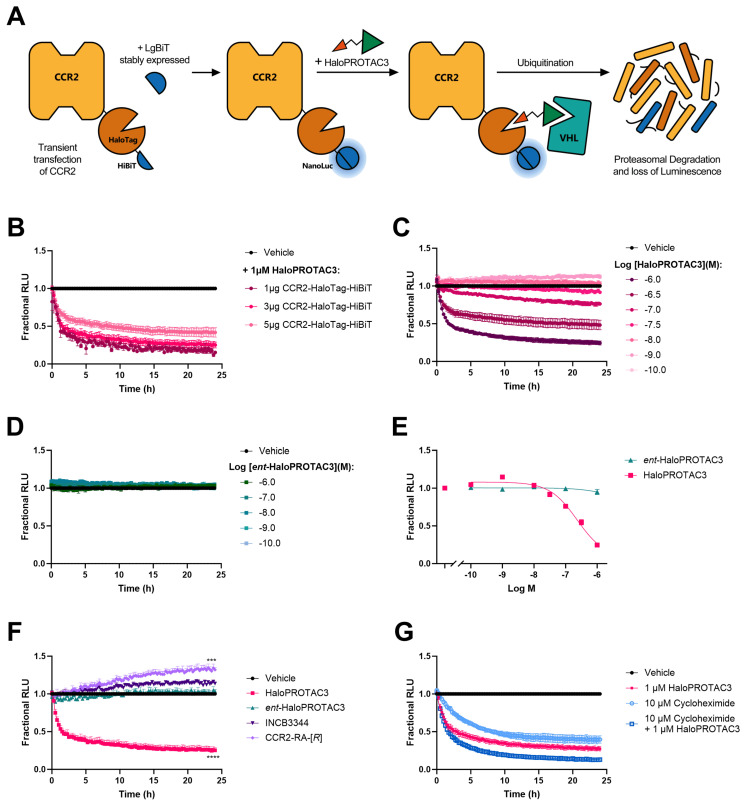
Real-time HiBiT detection assays to measure CCR2-HaloTag-HiBiT levels over 24 h, after treatment with indicated compounds. (**A**) Schematic drawing of the real-time HiBiT assays. HEK293-LgBiT cells were transfected with 3 µg of CCR2-HaloTag HiBiT (unless indicated otherwise), allowing the immediate complementation of HiBiT and LgBiT to form Nanoluciferase (NanoLuc). Transfected cells were then treated with selected compounds and luminescence, expressed as relative light units (RLUs), and continuously measured over 24 h. (**B**) Kinetic degradation profiles obtained in HEK293-LgBiT cells transfected with 1 µg, 3 µg, or 5 µg of CCR2-HaloTag HiBiT after treatment with 1 µM of HaloPROTAC3. (**C**,**D**) Kinetic degradation profiles of CCR2-HaloTag HiBiT after treatment with multiple concentrations of HaloPROTAC3 (**C**) or *ent*-HaloPROTAC3 (**D**). (**E**) Degradation curves showing the fractional RLU values corresponding to D_max_ at each concentration of the compound. D_max_ values were obtained from the real-times traces shown in (**C**,**D**). (**F**) Kinetic profiles of CCR2-HaloTag HiBiT after treatment with a single concentration (1 µM) of the indicated compounds. (**G**) Kinetic degradation profiles of CCR2-HaloTag HiBiT after treatment with 10 µM of cycloheximide or 1 µM of HaloPROTAC3 in the absence or presence of cycloheximide. In all cases, luminescence (RLU) was measured over 24 h in 15-min intervals. RLU values from mock-transfected HEK293-LgBiT cells were used for baseline-correction, and data were normalized to vehicle control. Data are shown as mean ± SEM of at least three independent experiments performed in triplicates. Statistical differences between fractional RLU values of compounds versus *ent*-HaloPROTAC3 (**F**) were analyzed using one-way ANOVA with Dunnett’s post-hoc test: *** *p* < 0.001, **** *p* < 0.0001.

To obtain real-time luminescence measurements, we used HEK293 cells stably expressing the LgBiT protein (HEK293-LgBiT). Transfection with CCR2-HaloTag-HiBiT results in immediate complementation with the endogenously expressed LgBiT protein, which allows the monitoring of luminescence in real time over a 24 h period and does not require cell lysis (Figure 3A). We transfected HEK293-LgBiT with 5 µg of CCR2-HaloTag-HiBiT treated cells with 1 µM of HaloPROTAC3, which resulted in a time-dependent decrease of CCR2 levels with maximal degradation of 60 ± 6%, as indicated by the reduction in luminescence at 24 h (Figure 3B). As the observed maximal degradation with 5 µg of plasmid was much higher in this real-time assay compared to the lytic assays, we tested whether initial CCR2 levels have an effect on maximal degradation in this assay. We repeated the time-course experiment with cells transfected with 3 µg or 1 µg of CCR2-HaloTag-HiBiT plasmid and treated the cells with 1 µM of HaloPROTAC3. Maximal degradation was further increased after 24 h of treatment to 76 ± 4% with 3 µg and 89 ± 2% with 1 µg of plasmid (Figure 3B). CCR2 levels remained unchanged after treatment with 1 µM of *ent*-HaloPROTAC3 among all DNA plasmid concentrations (Figure S5).

For further real-time experiments, we decided to continue with 3 µg of plasmid, as this provided a bigger degradation window compared to the 5 µg of plasmid while maintaining a similar maximal degradation compared to the lowest plasmid concentration. At 3 µg of plasmid concentration, HaloPROTAC3 (1 µM) maintained D_max_ for ~15 h, reaching ≥90% degradation after 9 h of treatment up to 24 h, without displaying protein recovery (Figure 3B,F). Furthermore, by fitting the degradation profile to a one-phase exponential decay equation, we calculated a degradation rate (*K*_deg_) of 0.52 ± 0.04 h^−1^ for CCR2-HaloTag-HiBiT by 1 µM of HaloPROTAC3. Of note, the degradation rates were similar among different plasmid concentrations, with a rate of 0.47 ± 0.04 h^−1^ at 1 µg of plasmid and 0.41 ± 0.17 h^−1^ at 5 µg of plasmid. D_max_ was also concentration-dependent, as shown by the decreasing D_max_ values with lower HaloPROTAC3 concentrations (Figure 3C), resulting in a degradation potency (pD_max50_) of 6.7 ± 0.1 (Figure 3E). In contrast, *ent*-HaloPROTAC3 did not change CCR2 levels at any of the concentrations tested (Figure 3D,E). We also tested the effect of the CCR2 antagonists, and in agreement with the results from the lytic assay format (Figure 2B,C), CCR2 levels increased over time by 20% and 50% when treated with 1 µM of INCB3344 or 1 µM of CCR2-RA-[*R*], respectively (Figure 3F).

To achieve degradation, a PROTAC needs to surpass the native degradation rate of its target protein. Thus, we explored the natural half-life of the CCR2-HaloTag-HiBiT construct by measuring the protein levels in the presence of the protein synthesis inhibitor cycloheximide. Treatment with 10 µM of cycloheximide for up to 24 h resulted in a fast reduction of CCR2 levels, characterized by a native degradation rate (*K*_native_) of 0.25 ± 0.01 h^−1^, and a half-life of 2.7 h (Figure 3G). However, the degradation rate of CCR2-HaloTag-HiBiT induced by 1 µM of HaloPROTAC3 in the absence or presence of cycloheximide was higher than that determined by cycloheximide on its own. This is apparent by the faster reduction in CCR2 levels and the higher D_max_ achieved with both HaloPROTAC3 and HaloPROTAC3 in the presence of cycloheximide (Figure 3G).

### 2.4. HaloPROTAC3 Induces Proteasomal Degradation of CCR2-HaloTag-HiBiT

**Figure 4:**
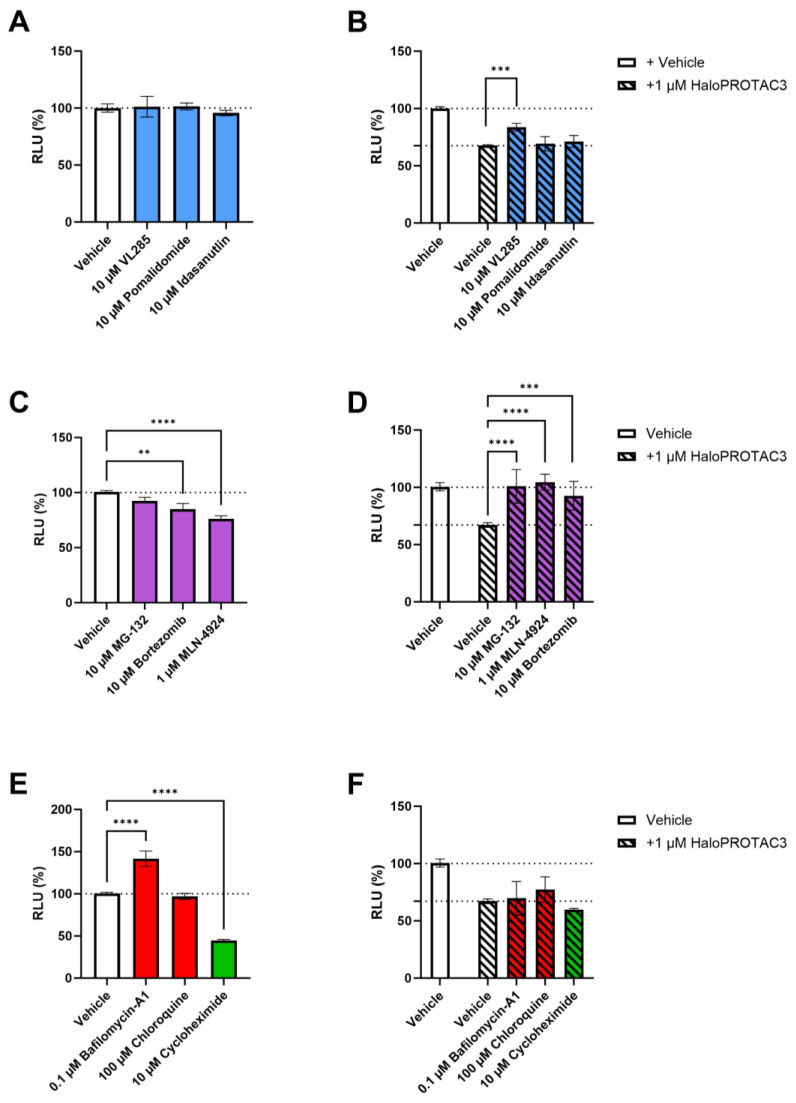
Lytic HiBiT detection assays to measure CCR2 levels in HEK293T cells transfected with 5 µg of CCR2-HaloTag HiBiT. Transfected cells were pretreated with the indicated inhibitors for 2 h before treatment with vehicle (**A**,**C**,**E**) or 1 µM of HaloPROTAC3 (**B**,**D**,**F**) for three more hours and measuring luminescence. E3 ligase inhibitors VL285, pomalidomide and idasanutlin are shown in blue (**A**,**B**); proteasomal (MG-132 and bortezomib) and neddylation (MLN-4924) inhibitors in magenta (**C**,**D**); lysosomal inhibitors bafilomycin-A1 and chloroquine in red; and the protein synthesis inhibitor cycloheximide in green (**E**,**F**). RLU values from mock-transfected cells were used for baseline-correction in all cases. Graphs show mean ± SEM values obtained from at least three independent experiments performed in triplicate. Statistical differences between normalized RLU values were analyzed using one-way ANOVA with Dunnett’s post-hoc test: ** *p* < 0.01, *** *p* < 0.001, **** *p* < 0.0001.

To investigate the mechanism of action of HaloPROTAC3-induced degradation of CCR2-HaloTag-HiBiT, we pretreated the cells with a variety of inhibitors. As HaloPROTAC3 comprises a VHL ligand (VL285) modified with a chloroalkane moiety, we first aimed to determine if the measured degradation was VHL-dependent. We pretreated the cells with three different reference E3 ligase inhibitors: a VHL ligand, VL285; a cereblon (CRBN) inhibitor, pomalidomide; and a mouse double minute 2 (MDM2) inhibitor, idasanutlin. After 2 h, the cells were treated with vehicle or HaloPROTAC3 (1µM) and degradation was measured after 3 h of HaloPROTAC3 treatment or vehicle control. Treatment with the E3 ligase inhibitors on their own did not affect receptor levels at the concentration tested (Figure 4A). In the presence of HaloPROTAC3, VL285 pretreatment significantly reduced the maximum CCR2 degradation to 16%, instead of 32% degradation in the absence of VL285, while neither pomalidomide nor idasanutlin had an effect on HaloPROTAC3-mediated degradation of CCR2-HaloTag-HiBiT. (Figure 4B).

Next, we explored whether degradation occurs via the proteasomal or the lysosomal pathway. To do so, we used the proteasomal inhibitors bortezomib and MG-132 or the lysosomal inhibitors bafilomycin-A1 and chloroquine. In addition, we also used the neddylation inhibitor MLN-4924 to explore whether degradation is dependent on the Cullin machinery. Finally, we included cycloheximide as an inhibitor of protein synthesis. Exclusive treatment with bortezomib and MLN-4929 led to ~17% and 24% reduction in CCR2 levels, respectively, while this was not observed with MG-132 (Figure 4C). Co-treatment with HaloPROTAC3 and proteasomal/neddylation inhibitors led to a full recovery of CCR2 levels (Figure 4D), indicating that HaloPROTAC3 induces degradation of CCR2-HaloTag-HiBiT via the proteasome. In contrast, exclusive treatment with Bafilomycin-A1 led to a >30% increase in CCR2-HaloTag-HiBiT levels, while receptor levels were not affected by chloroquine treatment (Figure 4E). In line with its effects in the kinetic assays (Figure 3G), cycloheximide reduced CCR2 levels by more than 50%, indicative of native degradation (Figure 4E). HaloPROTAC3-induced degradation was not affected when cells were pretreated with cycloheximide or any of the lysosomal inhibitors (Figure 4F), further confirming the role of the proteasome in HaloPROTAC3-mediated CCR2-HaloTag-HiBiT degradation. Thus, while the natural CCR2 turnover appears to rely on the lysosomal pathway, as shown by the treatment with Bafilomycin-A1 (Figure 4E), HaloPROTAC3 overrides the natural turnover and mediates degradation using the proteasomal pathway.

### 2.5. Immunofluorescence Studies Confirm CCR2-HaloTag-HiBiT Degradation by HaloPROTAC3

**Figure 5:**
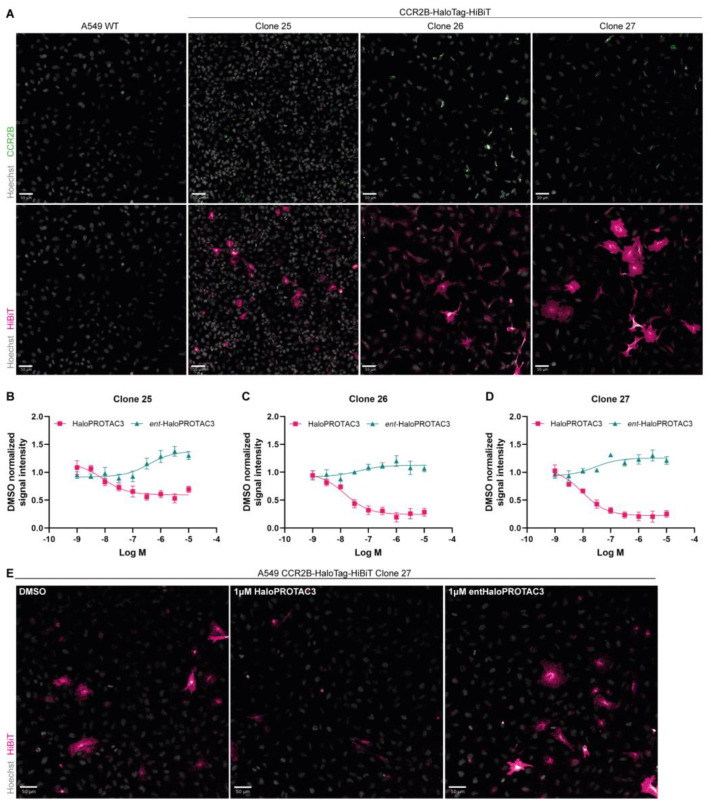
Immunofluorescence experiments to investigate CCR2-HaloTag-HiBiT degradation. (**A**) Immunofluorescence on A549 wild-type (WT) cells, or single-cell clones #25–27, expressing CCR2-HaloTag-HiBiT with antibodies staining for CCR2B (top row) or HiBiT (bottom row). Signals have been reduced based on A549 wild-type images to remove background, and all images have the same contrast and brightness. (**B**–**D**) Cells were treated for 24 h with increasing concentrations of HaloPROTAC3 or *ent*-HaloPROTAC3, and DMSO concentrations were adjusted. Cells were then imaged via immunofluorescence with an anti-HiBiT antibody and analyzed using Columbus for signal intensity. Shown are the degradation curves obtained from the normalized intensity data in each clone. Data are shown as mean ± SEM of four independent experiments performed in triplicates. (**E**) Representative images from (**D**) for DMSO, 1 μM HaloPROTAC3 or 1 μM *ent*-HaloPROTAC3. All scale bars show 50 μm.

While the HiBiT luminescence approach provides a good platform for high-throughput screening of targeted protein degradation, integral membrane protein degradation studies benefit from information about their localization. Hence, we performed immunofluorescence experiments to gain that additional level of information. In the first attempt, we used the transient transfection approach; however, residual DNA containing transfection complexes was co-stained with nuclei, preventing us from making batch image analysis. To mitigate the need for transfection, we transduced A549 cells with lentiviral particles for stable integration of expression cassettes for CCR2-HaloTag-HiBiT expression constructs, followed by antibiotic selection and limiting dilution to generate single-cell clones. We ended up with three single-cell clones: #25, #26, and #27. We validated those clones with an antibody specific for CCR2 isoform B (Figure 5A, top row), and an antibody against the HiBiT tag (Figure 5A, bottom row). The HiBiT antibody shows clear overexpression of the fusion protein in all three clones; however, expression within each clonal population varies from low to high signal intensity. Notably, the HiBiT signal appears throughout the cell, including the plasma membrane, with accumulated signal in the perinuclear/ER region (Figure 5A, bottom row). In contrast, the CCR2B-specific antibody produced only mildly higher signals in the expression clones than in the A549 WT cells, and these signals mostly clustered to the perinuclear/ER region (Figure 5A, top row). Thus, we decided to continue with the HiBiT antibody for further analysis, as it provided the highest signal.

Next, we used these stable expression cell lines to assess CCR2-HaloTag-HiBiT degradation using HaloPROTAC3 or the enantiomer. In clones #26 and #27, treatment with 1 µM of HaloPROTAC3 reduced CCR2-HaloTag-HiBiT levels by approximately 80% as determined by HiBiT antibody signals, whereas in clone #25, maximal degradation was 39% (Figure 5B–D). Despite the differences in maximal degradation, we found by fitting the degradation profile to a sigmoidal concentration-response curve that the DC_50_ was similar in all three clones, with 17.1 nM (pDC_50_ = 8.0 ± 0.3) in clone #25, 18.1 nM (pDC_50_ = 7.8 ± 0.1) in clone #26 and 11.8 nM ± (pDC_50_ = 8.0 ± 0.2) in clone #27. Degradation of CCR2-HaloTag-HiBiT led to an overall reduction of signals, with perinuclear signals remaining in the HaloPROTAC3 treated cells (Figure 5E, middle panel). In contrast, treatment with 1 µM of entHaloPROTAC3 appeared to increase the signal throughout the cell (Figure 5B–E, right panel).

### 2.6. Degradation of CCR2-HaloTag-HiBiT Translates to Reduced Receptor Activation

**Figure 6:**
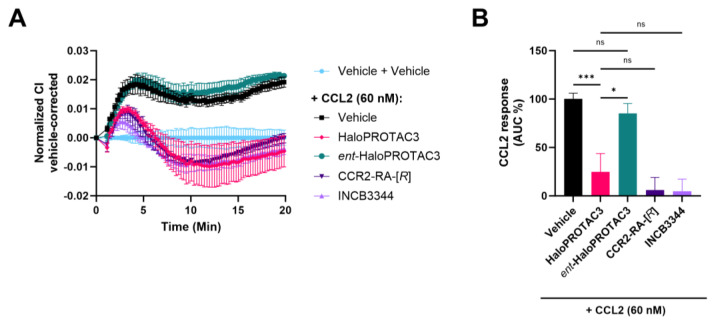
xCELLigence assays to investigate functional inhibition of CCR2-HaloTag-HiBiT after inducing degradation. (**A**) Representative vehicle-corrected, normalized Cell Index (CI) xCELLigence traces obtained with transfected HEK293T cells pretreated with the indicated compounds at 1 µM for 3 h before stimulation with 60 nM CCL2. Representative data are shown as mean ± SD of a single experiment performed in triplicate. (**B**) CCL2 cellular response was derived from the vehicle-corrected, normalized CI traces and is expressed as the area under the curve (AUC) within the first 20 min after stimulation. AUC values were normalized to the response of 60 nM CCL2 on its own for comparison. Data are shown as mean ± SEM of at least three independent experiments performed in triplicates. Statistical differences between normalized CCL2 responses were analyzed using one-way ANOVA with Tukey’s post-hoc test: ns, not significant, * *p* < 0.05, *** *p* < 0.001.

Finally, we aimed to investigate whether treatment with HaloPROTAC3 results in functional inhibition of CCR2 signaling. For this, we again used the label-free, impedance-based xCELLigence assay, which allow us to monitor cellular changes mediated by CCR2 signaling. HEK293T cells transiently transfected with CCR2-HaloTag-HiBiT were pretreated for 3 h with 1 µM of HaloPROTAC3, *ent*-HaloPROTAC3, and the CCR2 antagonists INCB3344 and CCR2-RA-[*R*] before stimulation with a submaximal concentration of CCL2 (60 nM). In agreement with our previous results (Figure 1C), stimulation with 60 nM CCL2 resulted in an increase in electrical impedance, as shown by the normalized CI values (Figure 6A). Near complete inhibition of CCL2-induced CCR2 activation was observed when pretreating the cells with the CCR2 antagonists INCB3344 and CCR2-RA-[*R*]. Analysis of the area under the curve over the first 20 min yielded a remaining response of 6 ± 13% with CCR2-RA-[*R*] and 5 ± 12% with INCB3344 (Figure 6A,B). Treatment with HaloPROTAC3 also resulted in inhibition of the cellular response induced by CCR2, as shown by the significant reduction in CCR2 activity (25 ± 19%, Figure 6A,B). Of note, there was no significant difference between the inhibition achieved by HaloPROTAC3 and the reference antagonists (Figure 6B). Compared to CCL2-mediated signaling, *ent*-HaloPROTAC3 did not affect the CCR2 response, as shown by the overlapping xCELLigence traces and a normalized response of 85 ± 10% (Figure 5A,B). Together, these data illustrate that HaloPROTAC3-induced degradation of CCR2-HaloTag-HiBiT results in functional inhibition of this tagged receptor.

## 3. Discussion

TPD has emerged as a novel concept to target a wide range of proteins; however, only a few GPCRs have been targeted in this manner. The few existing examples include the α_1A_-AR [13], GPER [14], and CCR9 [15]. Although these examples suggest the development of PROTACs as a feasible strategy to target GPCRs, the data presented in these studies is still limited. Importantly, these examples demonstrate that GPCR PROTACs can be designed to bind at either the extracellular or intracellular region of the receptor [17]. In this regard, multiple binding sites for small molecules have been described for CCR2, with some antagonists binding at the extracellular orthosteric binding site and others at an allosteric intracellular binding site [21,34]. Compounds binding at the intracellular site are particularly interesting for the development of PROTACs, as they can potentially link the intracellular interface of CCR2 with cytosolic E3 ligases. Thus, in this study, we decided to explore CCR2 degradation as a potential therapeutic avenue for this receptor.

To validate the feasibility of this approach for CCR2 without having to develop CCR2-specific PROTACs, we explored CCR2 degradation with the use of a tag-targeted degrader system. This allows the degradation of tagged proteins by using selective and cell-permeable degraders directed toward a protein tag [24,25,38], such as HaloTag7 [39]. Previous studies have used HaloTag7 fused to a variety of proteins localized in different subcellular compartments, such as the nucleus, endoplasmic reticulum, and lysosome [40]. Additionally, the GPCRs GPR40 and Frizzled-4 have been degraded using HaloTag7 or the highly similar HaloTag [41,42]. Other tag-targeted systems, like the dTAG system, have been used to explore the degradation of a variety of transmembrane solute carrier transporters [12]. In our case, we made use of the HaloTag7 protein tag to induce degradation [39], as well as the HiBiT tag to measure degradation via luminescence [31] (Figure 2A and Figure 3A). The addition of HaloTag7 to CCR2 did not seem to influence ligand binding (Figure 1B,C and Table 1) or receptor localization, as predominant signals were still in the perinuclear region and plasma membrane (Figure 5A), in line with previous reports [43]. However, it did alter receptor function in a label-free, real-time phenotypic assay (xCELLigence) [34,36], as CCL2 displayed a lower potency and efficacy in CCR2-HaloTag-HiBiT compared to CCR2-HiBiT (Figure 1E). Notably, the HiBiT tag on its own did not affect ligand binding or receptor functionality (Figure 1).

To induce degradation of HaloTag7-fusion proteins, several HaloPROTACs have been previously developed, which consist of a chloroalkane moiety that covalently binds to HaloTag7 and a VHL-targeting ligand [26,44]. Other than VHL, recent studies have described HaloPROTACs targeting other E3 ligases, such as CRBN [45] or cIAP1 [46,47]. In this study, we used HaloPROTAC3 (Figure S1), which selectively degrades HaloTag7-fusion proteins via recruitment of VHL [26,29]. HaloPROTAC3 was able to induce ~30–80% degradation of CCR2-HaloTag-HiBiT depending on the protein expression levels; in general, a higher degradation efficiency was measured with lower protein levels (Figure 2 and Figure 3). In this regard, previous studies have shown that overexpression due to transient transfection may lead to smaller degradation windows, slower degradation rates, and/or a reduced apparent potency [31,42]. These findings led us to investigate CCR2 degradation across different plasmid concentrations, but it is important to note that the exogenous expression of our fusion protein may have resulted in lower D_max_ values than with endogenous tagging of the target protein via CRISPR/Cas9 editing. In contrast to the observed degradation with HaloPROTAC3, receptor levels were significantly increased after treatment with different CCR2 antagonists (Figure 2 and Figure 3F). A similar increase in protein levels linked to inhibition has been reported for other proteins, such as BRD2 [31], indicating a potential compensatory mechanism on protein synthesis due to inhibition or a pharmacochaperone effect. Such compensatory mechanisms may be at play as well in CCR2, but further investigation is needed.

To investigate the mechanism of HaloPROTAC3-induced degradation of CCR2, we performed HiBiT assays in the absence or presence of several proteasomal and lysosomal inhibitors. We limited the incubation time with these inhibitors to 5 h in total (2 h preincubation plus 3 h in the presence of HaloPROTAC3) to avoid toxicity [48]. Based on the degradation kinetics of 1 µM of HaloPROTAC3, >70% degradation is already achieved after 3 h treatment (Figure 3B,F); thus, a 3 h incubation time should provide us with a significant degradation window to investigate the effect of the different inhibitors. Our results indicate that HaloPROTAC3 induces degradation using a VHL-, and proteasome-dependent mechanism. Indeed, CCR2 levels were partially or fully recovered in the presence of the VHL ligand VL285, as well as with the proteasomal inhibitors MG-132 and bortezomib (Figure 4B,D), while no change was observed when cells were treated with lysosomal inhibitors (Figure 4F). To further support a VHL-dependent mechanism, we also used the NEDD8-activating enzyme inhibitor MLN-4924, which prevents the conjugation of NEDD8 to VHL, rendering it inactive [49]. CCR2 degradation was also fully prevented in the presence of MLN-4924, indicating that active VHL is required (Figure 4D). Finally, our experiments with *ent*-HaloPROTAC3 (Figure S1), which does not recruit VHL [26,29], showed no degradation at any concentration tested (Figure 2, Figure 3 and Figure S5); on the contrary, the enantiomer exhibited slight stabilization of the fusion proteins in immunofluorescence (Figure 5B–E).

The half-life of the target protein is a crucial parameter to be considered when developing PROTACs or other targeted degradation strategies. In fact, the degree of PROTAC-mediated degradation is not only determined by the degradation rate of the PROTAC, but also by the ‘natural’ degradation rate of the target protein [50]. Thus, proteins with a short half-life, particularly those with a half-life of less than 2 h, are less susceptible to PROTAC-mediated degradation compared to long-lived proteins [50,51]. In the case of CCR2, previous studies have reported a short mRNA half-life of endogenous CCR2, ranging from 1.5 to 2.8 h in fresh monocytes or THP-1 cells, respectively [52,53]. Using the established translation inhibitor cycloheximide [54,55], we determined that CCR2-HaloTag-HiBiT has a half-life of 2.7 h (Figure 3F), which is in line with the previously determined values for mRNA. Protein modifications are known to be a source of half-life alterations; for example, a recent study found a significant half-life reduction for HiBiT-tagged proteins compared to their untagged versions [51]. In contrast, HaloTag7 did not appear to affect the half-life of a protein phosphatase PP2A [56], similar to our construct. All in all, it is crucial to determine the protein half-life of any fusion protein, particularly when exploring targeted degradation strategies.

Tag-targeted degrader systems are also useful to investigate the functional consequences of protein degradation [29]. For example, Caine et al. showed that degradation of β-catenin-HaloTag-HiBiT by HaloPROTAC3 resulted in a reduced response after stimulation of the Wnt signaling pathway [29]. Thus, we aimed to investigate if CCR2-HaloTag-HiBiT degradation also leads to functional inhibition of this tagged receptor by using an xCELLigence assay, which records changes in cell impedance as a measure of GPCR signaling [34,36]. In this assay, CCL2 induced an increase in electrical impedance in HEK293T cells expressing CCR2-HaloTag-HiBiT (Figure 6A,B). This CCL2-induced response is fully inhibited by treating the cells with CCR2 antagonists. Similarly, treatment with HaloPROTAC3 resulted in a diminished CCL2-mediated CCR2 activation (Figure 6A,B), which may be due to loss of cell surface localized protein, as seen in immunofluorescence (Figure 5). However, further studies are needed to determine the functional effect of CCR2 degradation in disease-relevant systems, including relevant cell lines with endogenous CCR2 expression or in vivo disease models, particularly when developing specific CCR2 chemical degraders such as CCR2 PROTACs. In this regard, CCR2 has been linked to a variety of immuno-inflammatory diseases, including cancer. In cancer, recent studies suggest that CCR2 is expressed not only in immune cells, such as monocytes and T cells but also in most types of solid and blood cancer cells, such as breast and renal carcinoma [8], which expands the applicability of future CCR2 degraders to a large variety of relevant cell lines and disease models.

In general, PROTACs promise several advantages over traditional inhibitors, including the potential for higher clinical efficacy due to the complete removal of the target protein [23], thus eliminating potential scaffolding functions of the target. Previous studies with transmembrane proteins have shown that targeted degradation can also be linked to functional and phenotypic outcomes, as shown by the examples of previously described PROTACs for the SLC9A4 transporter or GPER [12,14]. In the case of chemokine receptors, such as CCR2, it has been suggested that >90% receptor occupancy is needed to ensure sustained receptor inhibition and clinical efficacy [57,58]. As it is challenging to achieve such high occupancy levels with traditional inhibitors, especially if compensatory mechanisms are in place to further increase receptor levels or chemokine levels, PROTACs may represent a novel alternative to achieve sustained inhibition in the clinic. Indeed, previous studies have demonstrated that PROTACs can lead to sustained pharmacodynamic responses using ex vivo and in vivo assays [59]. Thus, the development of CCR2 PROTACs emerges as a promising novel strategy to target this receptor in the clinic; however, the functional effects of native CCR2 degradation still need to be confirmed in relevant in vitro and in vivo assays.

## 4. Materials and Methods

### 4.1. Materials

Tango™ CCR2-*bla* osteosarcoma (U2OS) cells modified to stably express the human CCR2B (U2OS-CCR2) were obtained from Invitrogen (Carlsbad, CA, USA); human embryonic kidney 293T (HEK293T) cells were obtained from the American Type Culture Collection (ATCC, Manassas, VA, USA); and HEK293 cells stably expressing the Large BiT protein (HEK293-LgBiT) were obtained from Promega (Madison, WI, USA). [^3^H]INCB3344 (specific activity 26 Ci mmol^−1^) and [^3^H]CCR2-RA-[*R*] (specific activity 60.6 Ci mmol^−1^) were custom-labeled by Vitrax (Placentia, CA, USA). VL285, Bortezomib (PS-341), Pevonedistat (MLN4924), MG-132, Chloroquine diphosphate, Bafilomycin A1 (Baf-A1) were all purchased from Selleck Chemicals (Bio-Connect, Huissen, The Netherlands); Cycloheximide from LKT Labs (St. Paul, MN, USA); and chemokine ligand CCL2 from PeproTech (Cranbury, NJ, USA). HaloPROTAC3, *ent*-HaloPROTAC3, NanoGlo^®^ HiBiT Lytic detection system, NanoGlo^®^ Endurazine™, and FuGENE6^®^ were obtained from Promega (Madison, WI, USA). Dulbecco’s modified Eagles medium (DMEM) was purchased from Sigma-Aldrich (Merck, Darmstadt, Germany) or Capricorn Scientific (Ebsdorfergrund, Germany). Hygromycin B Gold was purchased from InvivoGen (Toulouse, France). Bovine serum albumin (BSA, fraction V) was purchased from Sigma-Aldrich (Merck, Darmstadt, Germany). Pierce™ bicinchoninic acid (BCA) protein assay kit was purchased from ThermoFisher Scientific (Waltham, MA, USA).

The CCR2-HiBiT and CCR2-HaloTag-HiBiT fusion proteins were generated by Promega using standard cloning protocols. Human CCR2B (NP_001116868.1, UniProt accession number P41597-2) was cloned into pFC14K HaloTag^®^ CMV Flexi^®^ vector (Promega), resulting in a CCR2-HaloTag construct. A HiBiT tag was appended to this construct using a GGSSGGSSG linker between the HaloTag^®^7 and the HiBiT tag. In addition, human CCR2B was cloned into pFC37K HiBiT CMV-neo Flexi^®^ Vector (Promega), resulting in the CCR2-HiBiT, with the HiBiT tag attached to the C-terminus of CCR2B via a VSQGSSGGSSG linker sequence. Before use, the DNA sequence of the fusion proteins was verified using Sanger sequencing at Leiden Genome Technology Center (Leiden, The Netherlands).

### 4.2. Cell Culture

HEK293 cells were isolated from the kidney of a human embryo. HEK293T is a highly transfectable derivative of HEK293 containing the SV40 T-antigen. U2OS cells were isolated from a moderately differentiated sarcoma of the tibia of a 15-year-old white female. A549 is a non-small cell lung carcinoma isolated from the lung of a 58-year-old white male. HEK293T cells were cultured in 10 cm ø plates using Dulbecco’s Modified Eagle Medium (DMEM) high glucose (Capricorn, Ebsdorfergrund, Germany) supplemented with 10% fetal calf serum (FCS), 2 mM pf glutamine, 100 IU/mL of penicillin, and 100 µg/mL of streptomycin. HEK293-LgBiT cells were cultured in T75 flasks using the same medium as HEK293T cells, with the addition of 200 µg/mL of hygromycin B gold. Both cells were cultured for at least three passages before use in transfections. U2OS-CCR2 cells were cultured in 10 cm ø plates in McCoy’s 5A medium supplemented with 10% FCS, 2 mM of glutamine, 0.1 mM of non-essential amino acids, 25 mM of HEPES, 1 mM of sodium pyruvate, 100 IU/mL of penicillin, 100 µg/mL of streptomycin, 100 µg/mL of G418, 40 µg/mL of hygromycin B gold, and 125 µg/mL of zeocin. A549 cells and HEK293T cells for lentivirus generation were cultured in DMEM high glucose GlutaMAX (Gibco, ThermoFisher Scientific, Waltham, MA, USA) supplemented with 10% FCS (Corning, NY, USA). All cells were grown at 37 °C and 5% CO_2_.

### 4.3. Transfection and Lentivirus Generation

Before transfection, HEK293T or HEK293-LgBiT cells were seeded in 10 cm ø plates (2 × 10^6^ cells/plate) to reach ~50% confluence after 24 h. Cells were then transfected with 1 to 10 µg of CCR2 plasmid DNA using FuGENE6^®^ transfection reagent (Promega, Madison, WI, USA) in a 3:1 ratio of FuGENE6^®^ to DNA. The transfection mix was prepared in Opti-MEM™ I Reduced Serum Medium (Gibco, ThermoFisher Scientific, Waltham, MA, USA) and incubated for 15 min at room temperature before addition to the cells. Cells were further used in HiBiT assays or functional experiments 24 h after transfection or for membrane preparation 48 h after transfection. For lentivirus generation, HEK293T cells were seeded in a T25 flask (1.6 × 10^6^ cells) to reach 80% confluence after 24 h. The next day 3.75 µg of pPACK DNA (Cambridge Bioscience, Cambridge, UK) were transfected together with 3.75 µg of pCDH CMV CCR2B-HaloTag-HiBiT (synthesis and cloning by Geneart, Regensburg, Germany) using Mirus TransIT Lenti (Mirus, Madison, WI, USA), according to manufacturer’s protocol. Lentiviral particles were harvested after 48 h using a 0.45 µm pore size filter (Sartorius, Goettingen, Germany).

### 4.4. Transduction and Clonal Isolation

A549 cells were seeded at 400,000 cells per well in 6 well dishes to be approximately 50% confluent the next day. For seeding 1.5 mL of cell-containing medium was used. A total of 500 µL of lentiviral particles and 1.6 µL of polybrene (Sigma-Aldrich, Merck, Darmstadt, Germany) were added on top, and cells were incubated overnight. The next day, cells were washed two to three times in PBS and a growth medium containing 100 µg/mL of hygromycin B at 100 µg/mL was added. Cells were incubated until non-transduced cells were dead or until transduced cells were confluent (in which case they were split into a bigger flask in a hygromycin selection medium). After selection, cells were diluted to 0.5 cells/200 µL in a growth medium containing 20% FCS, and 96 well plates were seeded with 200 µL per well. Single-cell clones were grown back up to bigger populations and screened by immunofluorescence for HiBiT expression.

### 4.5. Membrane Preparation

For membrane preparation, U2OS-CCR2 cells were cultured in 15 cm ø plates using a medium of dialyzed FCS for at least one passage before harvesting the cells. In the case of membranes from HEK293T cells transfected with CCR2 constructs, cells were harvested 48 h after transfection. For harvesting, cells were scraped into 3–5 mL of phosphate-buffered saline (PBS) and centrifuged for 5 min at 3000 rpm. Membranes were separated from the cytosolic fractions by two homogenization and centrifugation steps. Pellets were resuspended in ice-cold Tris buffer (50 mM of Tris-HCl, 5 mM of MgCl2, pH 7.4), homogenized with an Ultra Turrax homogenizer (IKA-Werke GmbH & Co. KG, Staufen, Germany), and centrifuged at 31000 rpm for 20 min at 4 °C using an Optima LE-80K ultracentrifuge (Beckman Coulter, Inc., Fullerton, CA, USA). After the final centrifugation, the pellets were resuspended in ice-cold Tris buffer, aliquoted, and stored at −80 °C. Protein concentrations were determined using a standard Pierce™ BCA protein assay kit (ThermoFisher Scientific, Waltham, MA, USA).

### 4.6. Radioligand Binding Assays

Radioligand binding assays were performed using membranes from HEK293T cells transiently transfected with CCR2-HiBiT or CCR2-HaloTag-HiBiT, as well as membranes from U2OS-CCR2 cells. For the assays, membranes were homogenized and diluted in assay buffer (50 mM of Tris-HCl, 5 mM of MgCl_2_, 0.1% CHAPS, at pH 7.4) to achieve the desired concentration. For [^3^H]INCB3344, membranes from HEK293T cells containing CCR2-HaloTag-HiBiT were diluted to a final concentration of 20 µg per well; in all other cases, membranes were diluted to a final concentration of 10 µg per well in a final volume of 100 µL. Displacement assays were performed with ~5 nM of [^3^H]INCB3344 or ~6 nM of [^3^H]CCR2-RA-[*R*] and their unlabeled ligands at increasing concentrations, all diluted in assay buffer. Total binding was determined in the absence of ligand, while non-specific binding was determined by the addition of 10 µM of INCB3344 or CCR2-RA-[*R*], respectively. In all cases, final DMSO concentrations did not exceed 0.25%, and total binding did not exceed 10% of the amount of radioligand added in order to avoid ligand depletion. Reaction mixtures were incubated for 2 h at 25 °C while shaking, and incubations were terminated with ice-cold wash buffer (50 mM of Tris-HCl, 5 of mM MgCl_2_, 0.01% CHAPS, at pH 7.4) through a 96-well GF/C filter plate using a PerkinElmer Filtermate harvester (PerkinElmer, Groningen, The Netherlands). After washing and drying the filter, 25 µL of Microscint scintillation cocktail (PerkinElmer, Groningen, The Netherlands) was added to each well, and radioactivity was measured in a P-E 2450 Microbeta^2^ scintillation plate counter (PerkinElmer, Groningen, The Netherlands).

### 4.7. Label-Free Phenotypic Whole-Cell Assay (xCELLigence)

The xCELLigence Real-Time Cell Analyzer (RTCA) SP system (Agilent, Santa Clara, CA, USA) was used to measure CCL2-induced morphology changes of HEK293T cells transiently transfected with CCR2 constructs, using the impedance of electron flow as a readout. These functional assays were performed in 96-well E-plates PET (Agilent, Santa Clara, CA, USA) after 24 h of transfecting HEK293T cells with 5 µg of CCR2-HiBiT or CCR2-HaloTag-HiBiT. First, baseline-impedance was determined in 40 or 45 µL culture medium containing dialyzed FCS. Next, 50 µL of transfected cells were seeded at a density of 60,000 cells per well using culture medium with dialyzed FCS. After cell seeding, cells were allowed to settle in the E-plate for 30 min at room temperature before placing it in the xCELLigence station at 37 °C and 5% CO_2_. Impedance was then monitored every 15 min for 18–20 h until compound treatment. For stimulation assays, cells were stimulated with 5 µL of increasing concentrations of CCL2 or PBS as vehicle control. For degradation/inhibition studies, cells transfected with CCR2-HaloTag-HiBiT were pretreated for 3 h with 5 µL of HaloPROTAC3, *ent*-HaloPROTAC3, INCB3344, or CCR2-RA-[*R*], at a final concentration of 1 µM. As vehicle control, PBS supplemented with 0.1% DMSO was used during cell pretreatment. After 3 h, cells were simulated with a submaximal concentration of CCL2, corresponding to a final concentration of 60 nM of CCL2 or PBS as the vehicle. In all cases, compounds were added to the cells using a VIAFLO 96 handheld electronic 96-channel pipette (Integra Bioscience, Tokyo, Japan). After CCL2 stimulation, impedance changes were continuously recorded every 15 s for 25 min, followed by every minute for 10 min, every 5 min for 50 min, and finally every 15 min until stopping the experiment.

### 4.8. Lytic HiBiT Detection

After 24 h of transfecting HEK293T cells, mock-transfected and CCR2-transfected cells were seeded in solid, white 96-well tissue culture plates (Corning, NY, USA) at a density of 20,000 cells per well. Immediately after seeding, cells were treated with 10 µL of the indicated concentrations of various compounds for 24 h at 37 °C and 5% CO_2_. In the case of co-treatment experiments, cells were pretreated with 5 µL of proteasomal, lysosomal, or protein synthesis inhibitors at the indicated concentrations for 2 h before treatment with 5 µL of 1 µM of HaloPROTAC3 or vehicle control and further coincubation for three hours at 37 °C and 5% CO_2_. In all cases, 100 µL of NanoGlo^®^ HiBiT Lytic Reagent, containing a substrate and LgBiT protein, was added to each well after compound treatment and incubated in the dark for 20 min before measuring endpoint luminescence on a Flexstation 3 Multi-mode microplate reader (Molecular Devices, Wokingham, UK), using an integration time of 1 s.

### 4.9. Real-Time, Live Cell HiBiT Detection

After 24 h of transfecting HEK293T cells, mock-transfected and CCR2-transfected cells were seeded in clear-bottom, white 96-well tissue culture plates (Greiner Bio-One, Kremsmünster, Austria) at a density of 40,000 cells per well in a volume of 40 µL. For continuous luminescence detection over 24 h, 50 µL of NanoGlo^®^ Endurazine™ was added to each well, using a 50-fold dilution of the substrate. After substrate addition, the plate was incubated at 37 °C for 2.5 h to allow luminescence to equilibrate before compound addition. CO_2_-independent medium (Gibco, ThermoFisher Scientific, Waltham, MA, USA) was used to seed the cells and dilute all reagents and compounds. After addition of compound addition, luminescence was read from the bottom of the plate every 15 min for a 24 h period and integration time of 1 s on a Flexstation 3 Multi-mode microplate reader (Molecular Devices, Wokingham, UK) set at 37 °C. To avoid evaporation, plates were sealed with a breathable rayon film (VWR, Avantor, PA, USA), and the lid was retained when possible.

### 4.10. Immunofluorescence

A549 CCR2b-HALO-HiBiT expressing cells were seeded onto 384-well plates suitable for high-throughput microscopy (PerkinElmer, Groningen, The Netherlands; Corning, NY, USA) with a density ranging from 2000–4000 cells per well. The next day, plates were treated with different concentrations of HaloPROTAC3 or *ent*-HaloPROTAC3 using a Tecan D300e digital dispenser. After 24 h, a volume of 8% PFA equal to seeding volume was added to each well for 10–15 min to fix the cells. Cells were then washed three times in PBS and simultaneously permeabilized and blocked for an hour using a modified blocking buffer (0.1% Triton (*v*/*v*), 1% BSA (*w*/*v*) (Sigma, A7030, Merck, Darmstadt, Germany)). Primary antibodies were diluted to 1:500 (CCR2b specific, 16154-1-AP, Proteintech, Manchester, UK; HiBiT Monoclonal antibody, N7200, Promega, Madison, WI, USA) and incubated at 4 °C overnight. Plates were washed three times in PBS and incubated with secondary antibody mix (Donkey anti-Rabbit IgG (H + L) Highly Cross-Adsorbed Secondary Antibody, Alexa Fluor™ 488, A21206, 1:5000; Goat anti-Mouse IgG (H + L) Cross-Adsorbed Secondary Antibody, Alexa Fluor™ 647, A21235, 1:5000; Hoechst33342, H3570, 1:5000; Cell Mask Orange, C10045, 1:30,000 (Invitrogen, Carlsbad, CA, USA)) for 1–3 h and washed again three times in PBS. Plates were imaged on either Yokogawa CV7000 or CV8000 systems with appropriate settings for detection of each relevant channel. Images were analyzed using Columbus image analysis.

### 4.11. Data Analysis and Statistics

All data were analyzed using GraphPad Prism 9.0 (GraphPad Software Inc., San Diego, CA, USA), and unless stated otherwise, all data are shown as mean ± SEM of at least three independent experiments performed in duplicate or triplicate. The (p)IC_50_ values were obtained by non-linear regression analysis of radioligand displacement assays using a one-site competition model in Prism.

Cell index (CI) values obtained in xCELLigence experiments were normalized to the last timepoint before CCR2 stimulation using the RTCA software v2.0 (Agilent, Santa Clara, CA, USA). The resulting normalized CI (nCI) traces were further corrected by subtracting the vehicle response, and peak nCI values from vehicle-corrected traces were obtained within 20 min of compound addition. (p)EC_50_ and E_max_ values were obtained by fitting the peak nCI values to a non-linear regression three-parameter response model. For degradation/inhibition assays in xCELLigence assays, the net area under the curve (AUC) over the first 20 min after CCL2 stimulation was calculated for the vehicle-corrected traces.

RLU values obtained from lytic or real-time HiBiT assays were baseline-corrected using the RLU values from mock-transfected cells. Fractional RLU values were calculated from the resulting baseline-corrected values by normalizing all values at a given time point to the vehicle-control value. For lytic HiBiT assays, data are presented as % of vehicle-control; for real-time HiBiT assays, data are presented as fractional RLUs, which corresponds to the ratio between the baseline-corrected RLU values of a compound and the RLU values of the vehicle at any given time point. The (p)DC_50_/D_max50_/EC_50_ values were obtained by non-linear regression curve fitting into a sigmoidal concentration-response curve with a fixed Hill slope of 1, using the equation Y = Bottom + (Top–Bottom)/(1 + 10^(X−logIC^_50_^)^) for degradation curves and Y = Bottom + (Top–Bottom)/(1 + 10^(logEC^_50_^−X)^) for enhancement curves. To calculate the degradation rate (*K*) from real-time HiBiT assays, the degradation curves were fitted to a one-phase exponential decay model following the equation Y = (Y_0_ − Plateau)e^−*K*t^ + Plateau.

Statistical differences between normalized RLU values from HiBiT assays or normalized CCL2 responses from xCELLigence assays were analyzed using one-way ANOVA with Dunnett’s or Tukey’s post-hoc test, and with significant differences noted as * *p* < 0.05, ** *p* < 0.01, *** *p* < 0.001, or **** *p* < 0.0001.

## 5. Conclusions

In this study, we used the HaloPROTAC3 system as a proof-of-concept to explore potential CCR2 degradation by PROTACs. We show that HaloPROTAC3-mediated degradation of CCR2-HaloTag-HiBiT is proteasomal, rather than lysosomal, for native CCR2 turnover. Furthermore, degradation of CCR2 results in a measurable loss of CCL2-mediated CCR2 function in a label-free phenotypic assay. Overall, our results show that despite its short half-life, CCR2 is amenable to chemical degradation and that CCR2 degradation translates into functional inhibition of this receptor. Thus, the development of CCR2-PROTACs as a novel strategy in drug discovery is warranted in the near future.

## Figures and Tables

**Table 1:** Binding affinity of CCR2-RA-[*R*] and INCB3344 as determined with homologous radioligand binding assays.

	pIC_50_ ± SEM (IC_50_, nM) ^1^		
Compound	CCR2 ^2^	CCR2-HiBiT ^3^	CCR2-HaloTag-HiBiT ^3^
CCR2-RA-[*R*]	7.9 ± 0.03 (13)	7.8 ± 0.06 (17)	7.9 ± 0.08 (13)
INCB3344	8.2 ± 0.13 (6)	8.2 ± 0.13 (7)	8.1 ± 0.10 (8)
SD-24	8.0 ± 0.06 (10)	8.3 ± 0.05 (5)	8.2 ± 0.05 (6)

^1^ Data shown represent mean ± SEM of three experiments performed in duplicate. ^2^ Affinity determined in membranes from U2OS cells stably expressing CCR2. ^3^ Affinity determined in membranes from HEK293T cells transiently transfected with the indicated construct (CCR2-HiBiT or CCR2-HaloTag-HiBiT).

**Table 2:** Effect of HaloPROTACs and CCR2 antagonists on CCR2 levels, as determined with HiBiT lytic assays using HEK293T cells transiently transfected with CCR2-HaloTag-HiBiT.

Compound	pDC_50_/pEC_50_ ± SEM (DC_50_/EC_50_, nM) ^1,2^	D_max_ ± SEM (%) ^1,3^
HaloPROTAC3	7.4 ± 0.01 (42)	38 ± 5
*ent*-HaloPROTAC3	n.a. ^4^	−1 ± 4
INCB3344	8.4 ± 0.3 (6)	−20 ± 5
CCR2-RA-[*R*]	7.8 ± 0.1 (15)	−49 ± 5
SD-24	6.8 ± 0.1 (181)	−48 ± 6

^1^ Data shown represent mean ± SEM of at least three experiments performed in triplicate. ^2^ Apparent potency of degradation (DC_50_) or apparent potency of CCR2 accumulation (EC_50_). ^3^ Maximal degradation (D_max_) determined at 1 µM of the compound. Negative values represent an increase in CCR2 levels. ^4^ not applicable.

## Data Availability

The data presented in this article are available from the authors on reasonable request.

## Supplementary Materials

The following supporting information can be downloaded at: https://www.mdpi.com/article/10.3390/ijms25168984/s1.

## References

[1] Hauser A.S., Attwood M.M., Rask-Andersen M., Schiöth H.B., Gloriam D.E. (2017). Trends in GPCR drug discovery: New agents, targets and indications. Nat. Rev. Drug Discov..

[2] Sriram K., Insel P.A. (2018). G protein-coupled receptors as targets for approved drugs: How many targets and how many drugs?. Mol. Pharmacol..

[3] Lopez-Cotarelo P., Gomez-Moreira C., Criado-Garcia O., Sanchez L., Rodriguez-Fernandez J.L. (2017). Beyond chemoattraction: Multifunctionality of chemokine receptors in leukocytes. Trends Immunol..

[4] Viola A., Luster A.D. (2008). Chemokines and their receptors: Drug targets in immunity and inflammation. Annu. Rev. Pharmacol. Toxicol..

[5] Zivkovic L., Asare Y., Bernhagen J., Dichgans M., Georgakis M.K. (2022). Pharmacological Targeting of the CCL2/CCR2 Axis for Atheroprotection: A Meta-Analysis of Preclinical Studies. Arterioscler. Thromb. Vasc. Biol..

[6] Georgakis M.K., Bernhagen J., Heitman L.H., Weber C., Dichgans M. (2022). Targeting the CCL2-CCR2 axis for atheroprotection. Eur. Heart J..

[7] Lim S.Y., Yuzhalin A.E., Gordon-Weeks A.N., Muschel R.J. (2016). Targeting the CCL2-CCR2 signaling axis in cancer metastasis. Oncotarget.

[8] Fei L., Ren X., Yu H., Zhan Y. (2021). Targeting the CCL2/CCR2 axis in cancer immunotherapy: One stone, three birds?. Front. Immunol..

[9] Alabi S.B., Crews C.M. (2021). Major advances in targeted protein degradation: PROTACs, LYTACs, and MADTACs. J. Biol. Chem..

[10] Churcher I. (2018). Protac-induced protein degradation in drug discovery: Breaking the rules or just making new ones?. J. Med. Chem..

[11] Burslem G.M., Smith B.E., Lai A.C., Jaime-Figueroa S., McQuaid D.C., Bondeson D.P., Toure M., Dong H., Qian Y., Wang J. (2018). The advantages of targeted protein degradation over inhibition: An RTK case study. Cell Chem. Biol..

[12] Bensimon A., Pizzagalli M.D., Kartnig F., Dvorak V., Essletzbichler P., Winter G.E., Superti-Furga G. (2020). Targeted Degradation of SLC Transporters Reveals Amenability of Multi-Pass Transmembrane Proteins to Ligand-Induced Proteolysis. Cell Chem. Biol..

[13] Li Z., Lin Y., Song H., Qin X., Yu Z., Zhang Z., Dong G., Li X., Shi X., Du L. (2020). First small-molecule PROTACs for G protein-coupled receptors: Inducing α1A-adrenergic receptor degradation. Acta Pharm. Sin. B.

[14] Lu A.S., Rouhimoghadam M., Arnatt C.K., Filardo E.J., Salem A.K. (2021). Proteolytic Targeting Chimeras with Specificity for Plasma Membrane and Intracellular Estrogen Receptors. Mol. Pharm..

[15] Huber M.E., Toy L., Schmidt M.F., Vogt H., Budzinski J., Wiefhoff M.F.J., Merten N., Kostenis E., Weikert D., Schiedel M. (2022). A Chemical Biology Toolbox Targeting the Intracellular Binding Site of CCR9: Fluorescent Ligands, New Drug Leads and PROTACs. Angew. Chem. Int. Ed. Engl..

[16] Ruffilli C., Roth S., Rodrigo M., Boyd H., Zelcer N., Moreau K. (2022). Proteolysis Targeting Chimeras (PROTACs): A Perspective on Integral Membrane Protein Degradation. ACS Pharmacol. Transl. Sci..

[17] Keen A.C., Jorg M., Halls M.L. (2023). The application of targeted protein degradation technologies to G protein-coupled receptors. Br. J. Pharmacol..

[18] Schapira M., Calabrese M.F., Bullock A.N., Crews C.M. (2019). Targeted protein degradation: Expanding the toolbox. Nat. Rev. Drug Discov..

[19] Congreve M., Oswald C., Marshall F.H. (2017). Applying structure-based drug design approaches to allosteric modulators of GPCRs. Trends Pharmacol. Sci..

[20] Ortiz Zacarias N.V., Lenselink E.B., IJzerman A.P., Handel T.M., Heitman L.H. (2018). Intracellular receptor modulation: Novel approach to target GPCRs. Trends Pharmacol. Sci..

[21] Zheng Y., Qin L., Ortiz Zacarías N.V., de Vries H., Han G.W., Gustavsson M., Dabros M., Zhao C., Cherney R.J., Carter P. (2016). Structure of CC chemokine receptor 2 with orthosteric and allosteric antagonists. Nature.

[22] Guedeney N., Cornu M., Schwalen F., Kieffer C., Voisin-Chiret A.S. (2023). PROTAC technology: A new drug design for chemical biology with many challenges in drug discovery. Drug Discov. Today.

[23] Kostic M., Jones L.H. (2020). Critical Assessment of Targeted Protein Degradation as a Research Tool and Pharmacological Modality. Trends Pharmacol. Sci..

[24] Yesbolatova A., Tominari Y., Kanemaki M.T. (2019). Ligand-induced genetic degradation as a tool for target validation. Drug Discov. Today Technol..

[25] Grohmann C., Magtoto C.M., Walker J.R., Chua N.K., Gabrielyan A., Hall M., Cobbold S.A., Mieruszynski S., Brzozowski M., Simpson D.S. (2022). Development of NanoLuc-targeting protein degraders and a universal reporter system to benchmark tag-targeted degradation platforms. Nat. Commun..

[26] Buckley D.L., Raina K., Darricarrere N., Hines J., Gustafson J.L., Smith I.E., Miah A.H., Harling J.D., Crews C.M. (2015). HaloPROTACS: Use of Small Molecule PROTACs to Induce Degradation of HaloTag Fusion Proteins. ACS Chem. Biol..

[27] Nabet B., Roberts J.M., Buckley D.L., Paulk J., Dastjerdi S., Yang A., Leggett A.L., Erb M.A., Lawlor M.A., Souza A. (2018). The dTAG system for immediate and target-specific protein degradation. Nat. Chem. Biol..

[28] Bond A.G., Craigon C., Chan K.-H., Testa A., Karapetsas A., Fasimoye R., Macartney T., Blow J.J., Alessi D.R., Ciulli A. (2021). Development of BromoTag: A “bump-and-hole”–PROTAC system to induce potent, rapid, and selective degradation of tagged target proteins. J. Med. Chem..

[29] Caine E.A., Mahan S.D., Johnson R.L., Nieman A.N., Lam N., Warren C.R., Riching K.M., Urh M., Daniels D.L. (2020). Targeted Protein Degradation Phenotypic Studies Using HaloTag CRISPR/Cas9 Endogenous Tagging Coupled with HaloPROTAC3. Curr. Protoc. Pharmacol..

[30] Schwinn M.K., Machleidt T., Zimmerman K., Eggers C.T., Dixon A.S., Hurst R., Hall M.P., Encell L.P., Binkowski B.F., Wood K.V. (2018). CRISPR-Mediated Tagging of Endogenous Proteins with a Luminescent Peptide. ACS Chem. Biol..

[31] Riching K.M., Mahan S., Corona C.R., McDougall M., Vasta J.D., Robers M.B., Urh M., Daniels D.L. (2018). Quantitative Live-Cell Kinetic Degradation and Mechanistic Profiling of PROTAC Mode of Action. ACS Chem. Biol..

[32] Daniels D.L., Riching K.M., Urh M. (2019). Monitoring and deciphering protein degradation pathways inside cells. Drug Discov. Today Technol..

[33] Riching K.M., Mahan S.D., Urh M., Daniels D.L. (2020). High-throughput cellular profiling of targeted protein degradation compounds using HiBiT CRISPR cell lines. JoVE (J. Vis. Exp.).

[34] Zweemer A.J., Nederpelt I., Vrieling H., Hafith S., Doornbos M.L., de Vries H., Abt J., Gross R., Stamos D., Saunders J. (2013). Multiple binding sites for small-molecule antagonists at the CC chemokine receptor 2. Mol. Pharmacol..

[35] Ortiz Zacarías N.V., van Veldhoven J.P.D., Portner L., van Spronsen E., Ullo S., Veenhuizen M., van der Velden W.J.C., Zweemer A.J.M., Kreekel R.M., Oenema K. (2018). Pyrrolone derivatives as intracellular allosteric modulators for chemokine receptors: Selective and dual-targeting inhibitors of CC chemokine receptors 1 and 2. J. Med. Chem..

[36] Doornbos M.L.J., Heitman L.H., Shukla A.K. (2019). Chapter 11—Label-free impedance-based whole cell assay to study GPCR pharmacology. Methods Cell Biology.

[37] Scott C.W., Peters M.F. (2010). Label-free whole-cell assays: Expanding the scope of GPCR screening. Drug Discov. Today.

[38] Röth S., Fulcher L.J., Sapkota G.P. (2019). Advances in targeted degradation of endogenous proteins. Cell. Mol. Life Sci..

[39] Ohana R.F., Encell L.P., Zhao K., Simpson D., Slater M.R., Urh M., Wood K.V. (2009). HaloTag7: A genetically engineered tag that enhances bacterial expression of soluble proteins and improves protein purification. Protein Expr. Purif..

[40] Simpson L.M., Glennie L., Brewer A., Zhao J.-F., Crooks J., Shpiro N., Sapkota G.P. (2022). Target protein localization and its impact on PROTAC-mediated degradation. Cell Chem. Biol..

[41] Neklesa T.K., Tae H.S., Schneekloth A.R., Stulberg M.J., Corson T.W., Sundberg T.B., Raina K., Holley S.A., Crews C.M. (2011). Small-molecule hydrophobic tagging–induced degradation of HaloTag fusion proteins. Nat. Chem. Biol..

[42] Bondeson D.P., Mullin-Bernstein Z., Oliver S., Skipper T.A., Atack T.C., Bick N., Ching M., Guirguis A.A., Kwon J., Langan C. (2022). Systematic profiling of conditional degron tag technologies for target validation studies. Nat. Commun..

[43] Kredel S., Wolff M., Hobbie S., Bieler M., Gierschik P., Heilker R. (2011). High-content analysis of CCR2 antagonists on human primary monocytes. J. Biomol. Screen..

[44] Tovell H., Testa A., Maniaci C., Zhou H., Prescott A.R., Macartney T., Ciulli A., Alessi D.R. (2019). Rapid and reversible knockdown of endogenously tagged endosomal proteins via an optimized HaloPROTAC degrader. ACS Chem. Biol..

[45] Ody B.K., Zhang J., Nelson S.E., Xie Y., Liu R., Dodd C.J., Jacobs S.E., Whitzel S.L., Williams L.A., Gozem S. (2023). Synthesis and Evaluation of Cereblon-Recruiting HaloPROTACs. ChemBioChem.

[46] Tomoshige S., Hashimoto Y., Ishikawa M. (2016). Efficient protein knockdown of HaloTag-fused proteins using hybrid molecules consisting of IAP antagonist and HaloTag ligand. Bioorg. Med. Chem..

[47] Tomoshige S., Naito M., Hashimoto Y., Ishikawa M. (2015). Degradation of HaloTag-fused nuclear proteins using bestatin-HaloTag ligand hybrid molecules. Org. Biomol. Chem..

[48] Burslem G.M., Crews C.M. (2020). Proteolysis-Targeting Chimeras as Therapeutics and Tools for Biological Discovery. Cell.

[49] Brownell J.E., Sintchak M.D., Gavin J.M., Liao H., Bruzzese F.J., Bump N.J., Soucy T.A., Milhollen M.A., Yang X., Burkhardt A.L. (2010). Substrate-Assisted Inhibition of Ubiquitin-like Protein-Activating Enzymes: The NEDD8 E1 Inhibitor MLN4924 Forms a NEDD8-AMP Mimetic In Situ. Mol. Cell.

[50] Bartlett D.W., Gilbert A.M. (2022). Translational PK–PD for targeted protein degradation. Chem. Soc. Rev..

[51] Vetma V., Perez L.C., Eliaš J., Stingu A., Kombara A., Gmaschitz T., Braun N., Ciftci T., Dahmann G., Diers E. (2024). Confounding Factors in Targeted Degradation of Short-Lived Proteins. ACS Chem. Biol..

[52] Sica A., Saccani A., Borsatti A., Power C.A., Wells T.N., Luini W., Polentarutti N., Sozzani S., Mantovani A. (1997). Bacterial lipopolysaccharide rapidly inhibits expression of C–C chemokine receptors in human monocytes. J. Exp. Med..

[53] Xu L., Rahimpour R., Ran L., Kong C., Biragyn A., Andrews J., Devries M., Wang J.M., Kelvin D. (1997). Regulation of CCR2 chemokine receptor mRNA stability. J. Leukoc. Biol..

[54] Kao S.-H., Wang W.-L., Chen C.-Y., Chang Y.-L., Wu Y.-Y., Wang Y.-T., Wang S.-P., Nesvizhskii A.I., Chen Y.-J., Hong T.-M. (2015). Analysis of protein stability by the cycloheximide chase assay. Bio-Protocol.

[55] Miao Y., Du Q., Zhang H.-G., Yuan Y., Zuo Y., Zheng H. (2023). Cycloheximide (CHX) chase assay to examine protein half-life. Bio-Protocol.

[56] Merrill R.A., Song J., Kephart R.A., Klomp A.J., Noack C.E., Strack S. (2019). A robust and economical pulse-chase protocol to measure the turnover of HaloTag fusion proteins. J. Biol. Chem..

[57] Schall T.J., Proudfoot A.E. (2011). Overcoming hurdles in developing successful drugs targeting chemokine receptors. Nat. Rev. Immunol..

[58] Bot I., Ortiz Zacarias N.V., de Witte W.E., de Vries H., van Santbrink P.J., van der Velden D., Kroner M.J., van der Berg D.J., Stamos D., de Lange E.C. (2017). A novel CCR2 antagonist inhibits atherogenesis in apoE deficient mice by achieving high receptor occupancy. Sci. Rep..

[59] Mares A., Miah A.H., Smith I.E.D., Rackham M., Thawani A.R., Cryan J., Haile P.A., Votta B.J., Beal A.M., Capriotti C. (2020). Extended pharmacodynamic responses observed upon PROTAC-mediated degradation of RIPK2. Commun. Biol..

