# Supplementary material for: Inducing Receptor Degradation as a Novel Approach to Target CC Chemokine Receptor 2 (CCR2)
Source: Int J Mol Sci. 2024 Aug 18;25(16):8984. doi: 10.3390/ijms25168984 (PMC11354370; doi:10.3390/ijms25168984)
Supplement: Supplementary file 1 [file ijms-25-08984-s001.zip › ijms-3137914-supplementary.pdf]

Supplementary information

Inducing Receptor Degradation as a Novel Approach to Target CC Chemokine Receptor 2 (CCR2)

Natalia V. Ortiz Zacarías [1,*], Sascha Röth [2], Jeremy D. Broekhuis [1,3], Daan van der Es [1], Kevin Moreau [2] and Laura H. Heitman [1,3]

[1] Division of Medicinal Chemistry, Leiden Academic Centre for Drug Research (LACDR), Leiden University, 2333 CC, Leiden, The Netherlands; jeremybroekhuis@outlook.com (J.D.B.); d.van.der.es@lacdr.leidenuniv.nl (D.v.d.E.); l.h.heitman@lacdr.leidenuniv.nl (L.H.H.)
[2] Safety Innovation and PROTAC Safety, Clinical Pharmacology & Safety Sciences, R&D, AstraZeneca, Cambridge CB2 0AA, UK; sascha.roth@astrazeneca.com (S.R.); kevin.moreau@astrazeneca.com (K.M.)
[3] Oncode Institute, 2333 CC, Leiden, The Netherlands
* Correspondence: n.v.ortiz.zacarias@lacdr.leidenuniv.nl

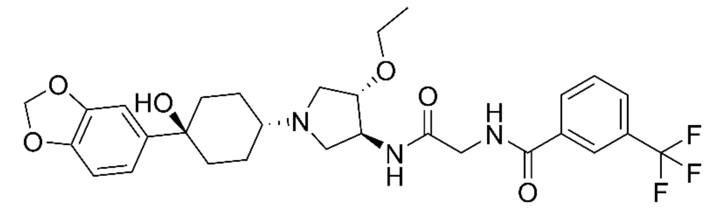

INCB3344

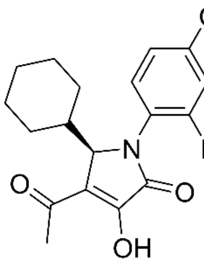

CCR2-RA-[*R*]

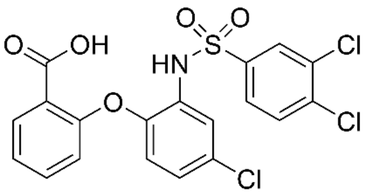

SD-24

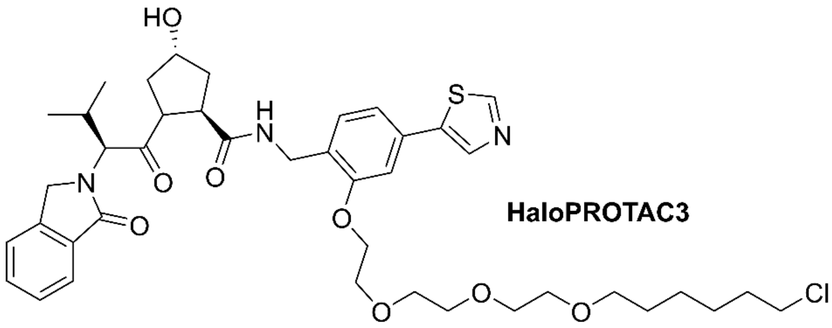

HaloPROTAC3

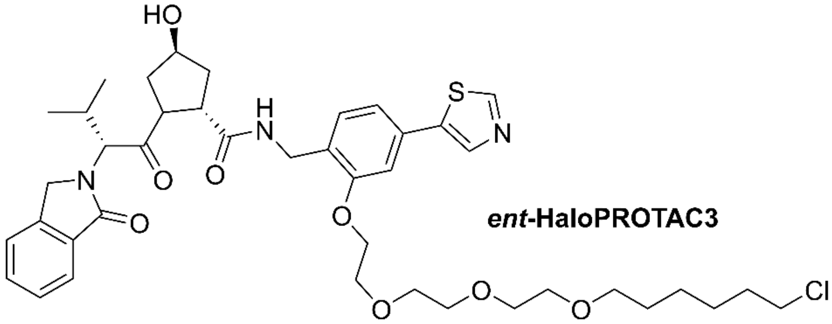

***ent*-HaloPROTAC3**

Figure S1. Chemical structures of the main compounds used in this study. Structures show the reference CCR2 antagonists INCB3344, CCR2-RA-[*R*] and SD-24; the chemical degrader HaloPROTAC3 and its enantiomer *ent*-HaloPROTAC3.

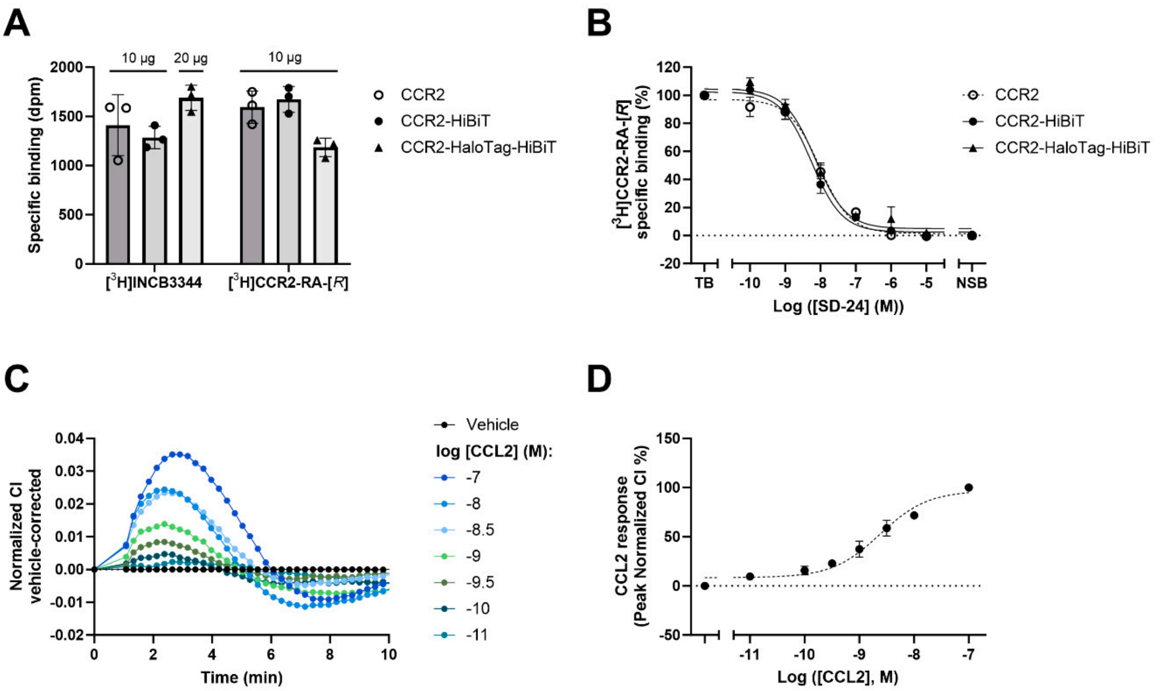

Figure S2. Pharmacological characterization of CCR2 wild-type and CCR2 constructs. (**A**) Specific [^{3}H]INCB3344 and [^{3}H]CCR2-RA-[*R*] binding in membranes from U2OS cells stably expressing CCR2 (referred as CCR2), or in membranes from HEK293T cells transiently transfected with CCR2-HiBiT or CCR2-HaloTag-HiBiT (referred as CCR2-HiBiT or CCR2-HaloTag-HiBiT in legend). Assays were performed with 10-20 µg of membranes and ~5 nM [^{3}H]INCB3344 or ~6nM [^{3}H]CCR2-RA-[*R*]. Specific binding are shown as mean ± SD disintegrations per minute (dpm) of three independent experiments performed in duplicate. (**B**) Displacement curves of [^{3}H]CCR2-RA-[*R*] specific binding by increasing concentrations of SD-24, in membranes from U2OS cells stably expressing CCR2, or from HEK293T cells transiently transfected with CCR2-HiBiT or CCR2-HaloTag-HiBiT. Radioligand binding data are shown as mean ± SEM of three independent experiments performed in duplicates. (**C**) Representative vehicle-corrected, normalized Cell Index (CI) traces measured in xCELLigence after stimulation of U2OS cells stably expressing CCR2 with vehicle (PBS) or increasing concentrations of CCL2. Data are shown as representative mean CI values of a single experiment performed in duplicate. (**D**) Combined concentration-response curve of CCL2 on U2OS cells stably expressing CCR2. CCL2 cellular response was derived from the vehicle-corrected, normalized CI traces, and is expressed as the maximum peak response within the first 6 min after stimulation. Data are shown as mean ± SEM of at least three independent experiments performed in duplicates.

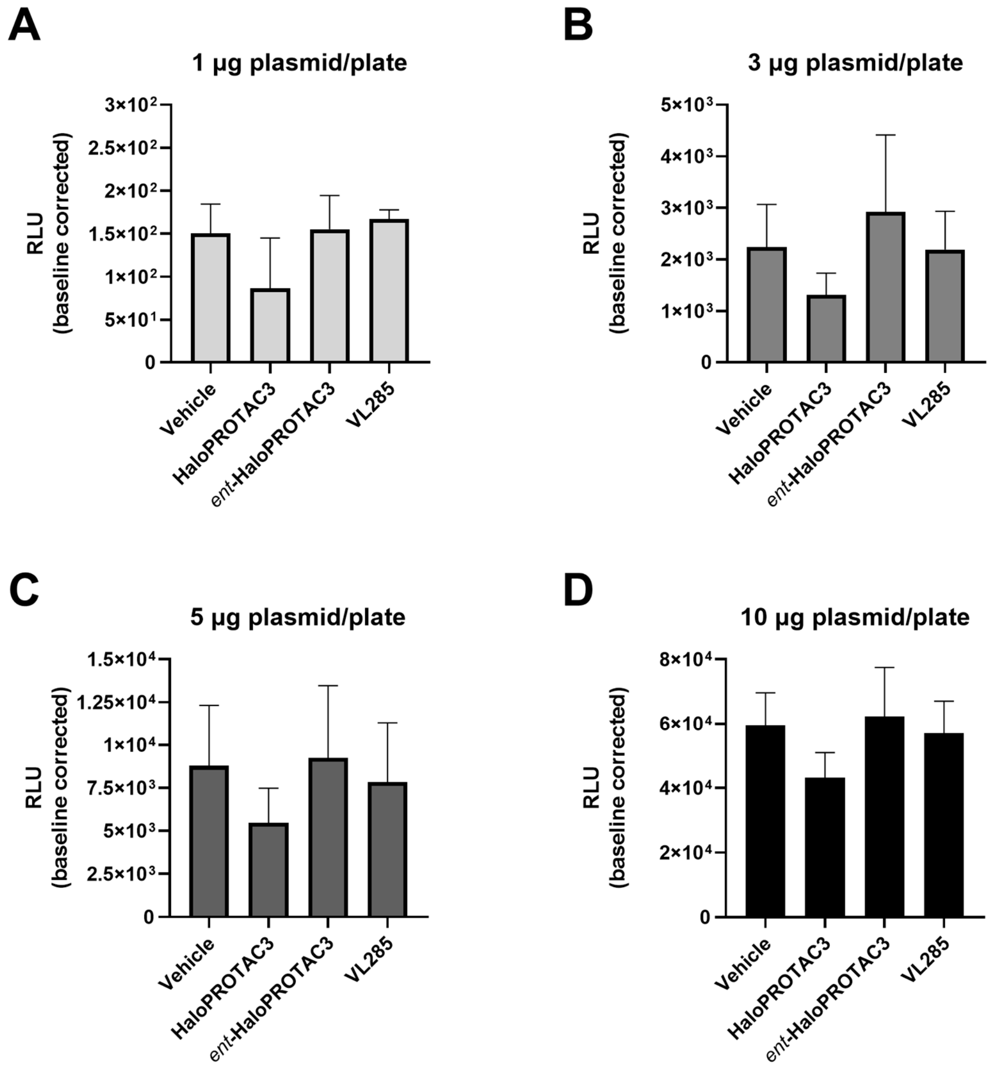

Figure S3. Lytic HiBiT Detection Assays to measure CCR2 levels. Assays were performed with HEK293T cells transfected with 1 µg (**A**), 3 µg (**B**), 5 µg (**C**), or 10 µg (**D**) of CCR2-HaloTag HiBiT. Transfected cells were treated with 1 µM HaloPROTAC3, *ent*-HaloPROTAC3, VL285 or vehicle control for 24h, before measurement of luminescence. RLU values from mock-transfected HEK293T cells were used for baseline-correction in all cases. Graphs show mean ± SD values obtained from two separate experiments performed in triplicate.

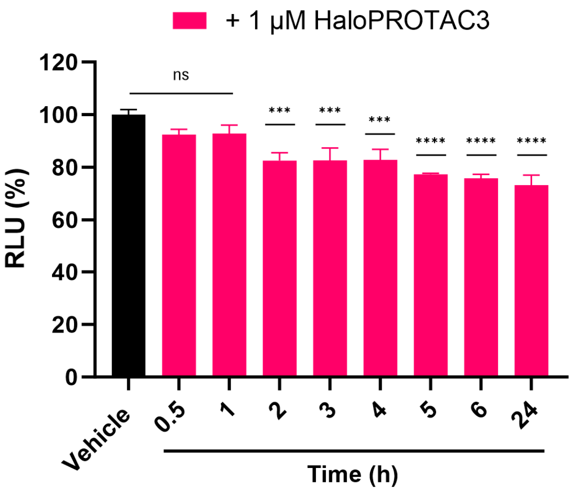

Figure S4. Lytic HiBiT Detection Assay to measure CCR2 levels over time. Assays were performed with HEK293T cells transfected with 5 μg CCR2-HaloTag HiBiT, and treated with 1 μM HaloPROTAC3 at the indicated time points before measurement of luminescence. RLU values from mock-transfected HEK293T cells were used for baseline-correction in all cases, and data were normalized to vehicle control. Data shown are mean ± SEM of three independent experiments performed in triplicates, and statistical analysis was performed using one-way ANOVA with Dunnett's post-hoc test (timepoints versus vehicle control): ***p<0.001, ****p<0.0001.

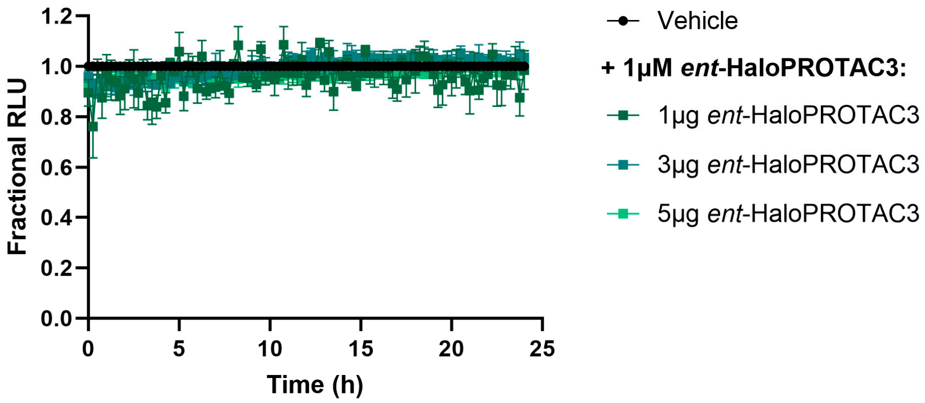

Figure S5. Effect of *ent*-HaloPROTAC3 on CCR2 levels over 24h, using real-time HiBiT detection assays. HEK293-LgBiT cells were transfected with 1, 3 or 5 μg of CCR2-HaloTag HiBiT, and treated with 1 μM of *ent*-HaloPROTAC3 just before measurement of luminescence over 24h in 15-min intervals. RLU values from mock-transfected HEK293-LgBiT cells were used for baseline-correction, and data were normalized to vehicle control. Data are shown as mean ± SEM of at least three independent experiments performed in triplicates.
